# Projected Lifetime Healthcare Costs Associated with HIV Infection

**DOI:** 10.1371/journal.pone.0125018

**Published:** 2015-04-22

**Authors:** Fumiyo Nakagawa, Alec Miners, Colette J. Smith, Ruth Simmons, Rebecca K. Lodwick, Valentina Cambiano, Jens D. Lundgren, Valerie Delpech, Andrew N. Phillips

**Affiliations:** 1 Research Department of Infection and Population Health, UCL, London, United Kingdom; 2 Department of Health Services Research and Policy, London School of Hygiene & Tropical Medicine, London, United Kingdom; 3 HIV and STI Department, Public Health England, London, United Kingdom; 4 Research Department of Primary Care and Population Health, UCL, London, United Kingdom; 5 Copenhagen HIV Programme (CHIP), Department of Infectious Diseases (8632), Rigshospitalet, University of Copenhagen, Copenhagen, Denmark; UNAIDS, TRINIDAD AND TOBAGO

## Abstract

**Objective:**

Estimates of healthcare costs associated with HIV infection would provide valuable insight for evaluating the cost-effectiveness of possible prevention interventions. We evaluate the additional lifetime healthcare cost incurred due to living with HIV.

**Methods:**

We used a stochastic computer simulation model to project the distribution of lifetime outcomes and costs of men-who-have-sex-with-men (MSM) infected with HIV in 2013 aged 30, over 10,000 simulations. We assumed a resource-rich setting with no loss to follow-up, and that standards and costs of healthcare management remain as now.

**Results:**

Based on a median (interquartile range) life expectancy of 71.5 (45.0–81.5) years for MSM in such a setting, the estimated mean lifetime cost of treating one person was £360,800 ($567,000 or €480,000). With 3.5% discounting, it was £185,200 ($291,000 or €246,000). The largest proportion (68%) of these costs was attributed to antiretroviral drugs. If patented drugs are replaced by generic versions (at 20% cost of patented prices), estimated mean lifetime costs reduced to £179,000 ($281,000 or €238,000) and £101,200 ($158,900 or €134,600) discounted.

**Conclusions:**

If 3,000 MSM had been infected in 2013, then future lifetime costs relating to HIV care is likely to be in excess of £1 billion. It is imperative for investment into prevention programmes to be continued or scaled-up in settings with good access to HIV care services. Costs would be reduced considerably with use of generic antiretroviral drugs.

## Introduction

Numerous studies have shown that effective antiretroviral therapy (ART) has reduced rates of HIV-related mortality[1, 2] and that life expectancy of people living with HIV has increased[3, 4]. Combined with the fact that there were 6,000 new cases of HIV diagnosed in the UK in 2013, of which a record high of 3,250 were in men-who-have-sex-with-men (MSM), the number of people requiring care is likely to rise further. Currently there are approximately 81,500 people accessing HIV care in the UK; a figure which has more than doubled in the last decade[5]. While HIV can now generally be successfully treated, it represents a burden for the HIV-positive individual and a major cost for healthcare services. Adoption of any ‘treatment as prevention’ policies, specifically earlier ART initiation, would add further costs per person with HIV. It would thus be useful to quantify the average healthcare costs that could be avoided by preventing HIV infections.

We have previously developed a stochastic computer simulation model of HIV progression and the effect of ART (HIV Synthesis) and used it to reconstruct the HIV-positive population in the UK and to predict future trends in key outcomes[6, 7]. In addition, by extending the length of projections made, it was also used to predict the life expectancy of MSM infected with HIV in 2010, assuming current standards of care remained[4]. In this study we build on our previous work to calculate the projected lifetime HIV-related healthcare cost for an individual with HIV infection, to estimate the costs that could be averted by preventing a single case of HIV. We also investigate the extent to which these lifetime costs could be reduced if patented drugs are replaced by generic antiretroviral drugs.

## Methods

### HIV Synthesis model

The HIV Synthesis progression model has been described previously[4, 6, 7] and has been recently updated. Details of the model and modifications can be seen in **S1 File**. In brief, it is an individual-based stochastic computer simulation model that generates simulated “data” on the progression of HIV infection and effect of ART on simulated individuals. For each simulated person, the model generates variables such as CD4 count, viral load, age, clinical events, use of specific antiretroviral drugs, resistance and adherence, all of which are updated in 3-month intervals. The model incorporates current estimated rates of virologic response to ART and subsequent long-term increases in CD4 counts. This version of the model does not include transmission so simulates HIV-positive individuals only. The model has been shown to provide a generally close fit to observed data relating to the natural progression and treatment outcomes[4, 6].

### Model assumptions

In the base-case analysis, we consider a scenario for a 30 year old MSM infected with drug-sensitive HIV in 2013. Outcomes were generated for 10,000 simulated people in this situation resulting in a distribution of possible outcomes for such a person. Each person was followed over 80 years (to 2093) or until death and it was assumed that they were never lost from care at any stage in life. It was further assumed that current standards of healthcare management are maintained in future years and that associated costs remain as now. Age-specific mortality rates for England and Wales for 2011 were used to calculate non-HIV related death rates[8]. We also assumed a potential raised risk of magnitude 1.5-fold (compared with UK population death rates) for non-AIDS deaths, due to the increasing evidence that presence of HIV is associated with a raised risk of common clinical conditions including renal, liver and cardiovascular diseases and non-AIDS cancers[9, 10]. This increased risk of non-AIDS death was assumed to be present regardless of viral load and CD4 count.

The rate of HIV diagnosis was chosen to reflect what has been observed recently in the UK for MSM in terms of CD4 count at diagnosis, i.e. median CD4 count of 422 cells/mm^3^ and 35% diagnosed late (CD4 count <350 cells/mm^3^ within 3 months of diagnosis) in 2011[11]. In undiagnosed people, it is assumed that development of an AIDS condition will also lead to diagnosis of HIV. Initiation of ART is modelled according to the 2012 British HIV Association (BHIVA) guidelines: first-line regimen consisting of a nucleoside backbone with either efavirenz, ritonavir-boosted atazanavir, ritonavir-boosted darunavir or raltegravir, started when the CD4 count drops below 350 cells/mm^3^ (unless symptomatic). Individuals switch to further lines of therapy if virologic failure occurs but can also switch individual drugs for toxicity. Boosted protease inhibitor (bPI)-based regimens are modelled to be double-potency compared to non-nucleoside reverse transcriptase inhibitor (NNRTI)-based regimens (i.e. acquisition of resistance mutations will impact suppression rates less on a bPI-based regimen). We assumed that 40% of MSM were smokers for life as observed[12, 13] and incorporated a two-fold higher rate of all-cause mortality associated with smoking[14]. It is also assumed that no one injects drugs and no one is co-infected with hepatitis viruses given the low rates observed in MSM in the UK[15,16].

### Costs

In this study we consider only costs associated with HIV-related healthcare. Costs for hospital or clinic services (hereafter referred to as healthcare centre visits) per 3-month period were stratified by CD4 count category (>200 and ≤200 cells/mm^3^) to fit closely with the available data[17]. These costs are assumed to include all inpatient and outpatient costs as well as costs relating to treatment of events such as clinical AIDS, AIDS defining diseases, symptomatic AIDS and toxicities resulting from ART-use. Other fixed unit costs used for each occurrence were CD4 count measures, viral load measures, genotypic resistance tests and ART-use (drug-specific costs). CD4 count and viral load measures were assessed 3-monthly and genotypic resistance tests were performed before ART initiation and at virologic failure. We assumed that healthcare centre visit costs incurred while someone is undiagnosed are the same as those of someone who is diagnosed but with a CD4 count >200 cells/mm^3^, on the basis that HIV infection raises the risk of common clinical conditions as mentioned previously (**Table 1**). The predicted average number of occurrences during a lifetime of each event listed above was multiplied by the unit cost (assumed to remain constant over time, except for discounting) and then summed to give an estimate of the cost of treating one HIV-positive individual for a lifetime. All costs (2013 UK pounds) were obtained from published sources (**Tables 1 and 2**) and discounted by 3.5% per annum in accordance with NICE (National Institute for Health and Care Excellence) recommendations. The presented lifetime costs also include costs of non-AIDS diseases in situations where the individual is seen in the same healthcare centre as for their HIV care.

**Table 1:** HIV-related care costs used in model.

Variable		Unit cost	Cost per person-year	Reference
Use of healthcare centre services (inpatient, outpatient and day ward)	Undiagnosed	-	£630^a^	[17]
	CD4 count >200 cells/mm^3^	-	£630^a^	[17]
	CD4 count ≤200 cells/mm^3^	-	£1430^a^	[17]
CD4 count assay		£34	-	[18]
Viral load assay		£63	-	[18]
Resistance test	Reverse transcriptase and protease sequencing	£219	-	[18]

^a^ these costs were derived using weighted averages from original costs found in reference [17]

**Table 2:** Costs of individual antiretroviral drugs used in model (assuming standard adult doses); taken from the British National Formulary.

Drug name	Cost per person-year, £
Abacavir	2,544
Didanosine	1,678
Emtricitabine	1,991
Lamivudine	1,831
Stavudine	1,937
Tenofovir	2,928
Zidovudine	2,100
Zidovudine (generic)	1,680
Kivexa (Abacavir+Lamivudine)	4,289
Trizivir (Abacavir+Lamivudine+Zidovudine)	6,198
Truvada (Emtricitabine+Tenofovir)	5,095
Combivir (Zidovudine+Lamivudine)	3,654
Atazanavir	3,694
Darunavir	5,439
Fosamprenavir	3,153
Kaletra (Ritonavir-boosted Lopinavir)	3,475
Ritonavir (as 100mg booster)	237
Efavirenz	2,438
Nevirapine	2,076
Etravirine	3,668
Atripla (Efavirenz+Emtricitabine+Tenofovir)	7,633
Enfuvirtide	13,171
Maraviroc	6,321
Raltegravir	6,377

These costs are only an approximate guide to what is actually paid in practice by local health authorities in the UK.

### Generic antiretroviral drugs

In addition to the base-case analysis, we also evaluated the lifetime costs assuming that generic antiretroviral drugs (generics) would replace patented versions approximately three years after patent expiry. We assumed that generics would be priced at an 80% reduction[19] of patented drugs. Drugs which are part of fixed-dose combinations are replaced individually as each patent expires. In reality, this would mean that instead of a once-daily pill combining three antiretrovirals, it would mean one fixed-dose pill combining two antiretrovirals and another pill for the generic. In this scenario, drug adherence is assumed to remain the same and would therefore not impact on regimen efficacy.

### Alternative strategies modelled

We also investigated the impact on lifetime costs of four alternative strategies to reduce HIV treatment and care costs[20]. In one strategy, CD4 count measures were taken annually instead of 3-monthly in individuals with suppressed viral load (<50 copies/ml) and with most recent CD4 count >350 cells/mm^3^. Measured CD4 counts in the model are used only to determine the likelihood of ART initiation or regimen switch after virologic failure (with an assumption of more rapid switch in those virologically failing with low CD4 count), and also whether PCP prophylaxis is used. In another strategy, we modelled all virologically suppressed individuals with no history of virologic failure to switch to ritonavir-boosted darunavir (DAR/r) mono-therapy. In this strategy, individuals start with standard triple-therapy (cART) regimens and if they fail the DAR/r-regimen after switching, they were modelled to switch back to an effective cART regimen. Finally, we also investigated the effect of 6-monthly and annual healthcare centre visits such that individuals attend clinics less and therefore have fewer CD4 count and viral load measures, but only if they have suppressed viral load, no history of virologic failure and with most recent CD4 count >350 cells/mm^3^_,_

### Sensitivity analyses

Univariable sensitivity analyses were performed to explore the effects of varying key assumptions on the overall lifetime costs and its various components. Assumptions which were varied include the age at infection, rate of diagnosis, rate of loss to care, ART initiation criteria, risk of non-AIDS deaths and population distribution of adherence. A multivariable sensitivity analysis was also conducted where the model was run 10,000 times, each time sampling values randomly from a distribution for different key parameters, to ascertain the variability and uncertainty associated with the estimates of lifetime costs.

## Results

For the 10,000 simulated people, median (interquartile range, IQR) duration of time before diagnosis was 2.5 (1–4.8) years. Median (IQR) CD4 count at diagnosis was 422 (247–568) cells/mm^3^ and 38% had CD4 count <350 cells/mm^3^ at diagnosis. Under the assumption that cART was initiated when the CD4 count dropped below 350 cells/mm^3^, the mean time spent from infection to treatment initiation was 5.2 years. Most (97%) people started first-line, 51% started second-line and 25% started third-line in their lifetime. Median CD4 count at time of starting ART was 301 cells/mm^3^. This was followed by a mean 19.4, 6.9 and 4.4 years spent on first-line, second-line, and third-line regimens and beyond respectively. At least one occurrence of treatment interruption occurred in 83% and of those who did interrupt, the mean total length of time spent off ART was six months. These outcomes led to a median (IQR) life expectancy of 71.5 (45.0–81.5) years.

**Fig 1:**
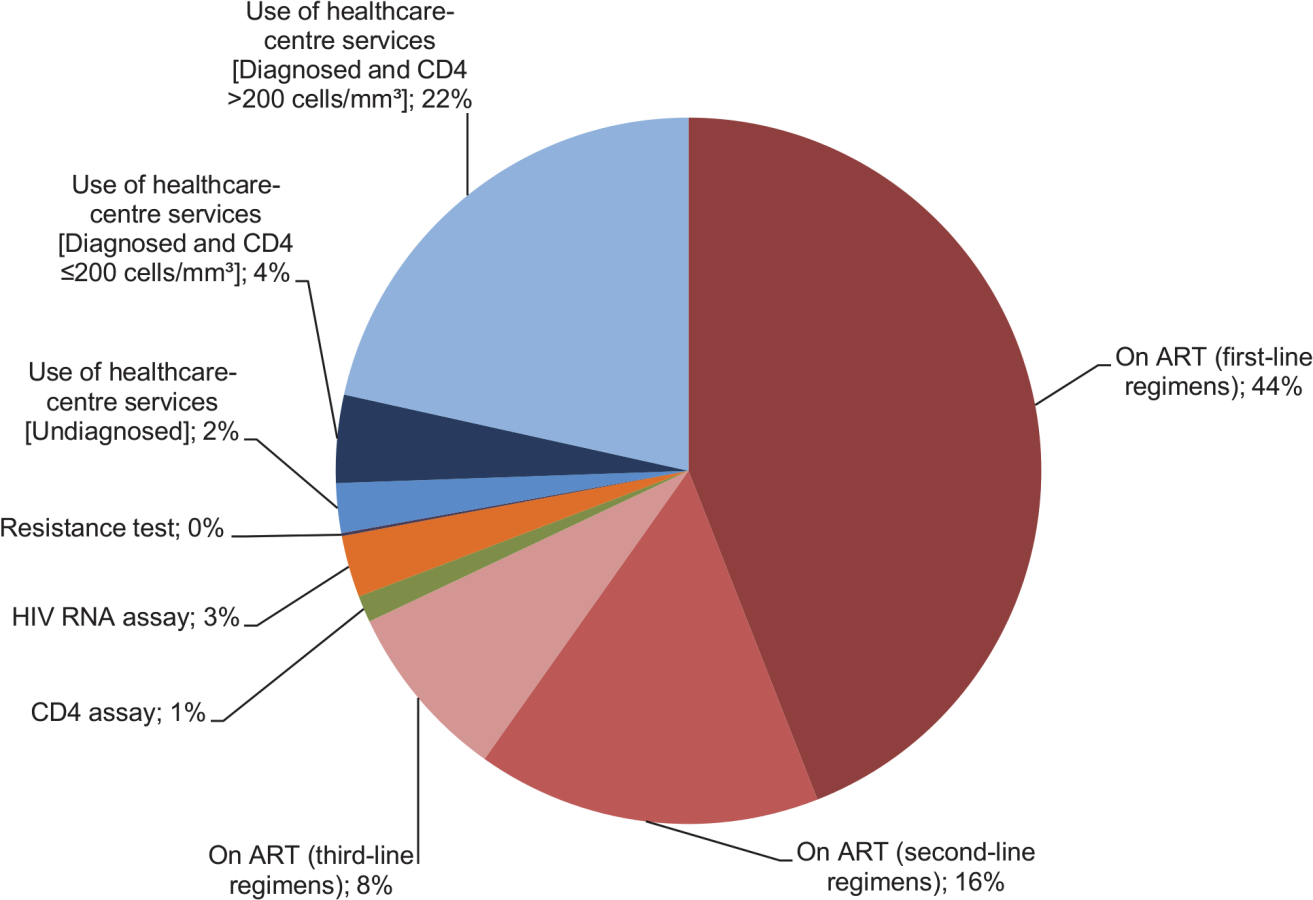
Distribution of costs spent in an average lifetime in base case analysis.

The estimated mean lifetime cost was £360,800 and with discounting at 3.5% per annum, it was £185,200(**Table 3**). The majority, 68% (£245,200), of projected lifetime healthcare cost was attributed to ART costs(**Fig 1**). Despite antiretroviral drugs used in second- and third-line regimens being more expensive in general, the average total undiscounted cost of first-line regimens at £158,900, was greater than subsequent lines (£56,900 and £29,500 on second- and third-line regimens) due to the longer time spent on first-line. Similarly, due to the longer time spent in the category ‘undiagnosed, or diagnosed and with higher CD4 counts’, compared to ‘diagnosed but with lower CD4 counts’, the costs incurred when in the former category are greater over a lifetime despite the lower costs incurred per person-year.

**Table 3:** Mean lifetime costs.

Scenario/strategy	Mean lifetime costs (2013 £)	Discounted at 3.5% (2013 £)	Reduction in cost from base-case analysis, %
Base-case analysis	360,800	185,200	-
Patented drugs replaced by generic versions (80% reduction in price)	179,600	101,200	Antiretroviral drug costs reduced by 74%
Yearly (instead of 3-monthly) CD4 count monitoring in individuals with suppressed viral load and most recent CD4 count >350 cells/mm^3^	357,500	183,800	CD4 count measurement costs reduced by 57%
Switch to DAR/r mono-therapy in virologically suppressed individuals who have never failed virologically before	330,600	169,200	Antiretroviral drug costs reduced by 12%
6-monthly (instead of 3-monthly) healthcare centre visits in individuals with suppressed viral load, most recent CD4 count >350 cells/mm^3^ and no history of virologic failure	344,000	176,500	Healthcare centre visit costs reduced by 20%
Yearly (instead of 3-monthly) healthcare centre visits in individuals with suppressed viral load, most recent CD4 count >350 cells/mm^3^ and no history of virologic failure	334,900	171,500	Healthcare centre visit costs reduced by 37%

DAR/r: ritonavir-boosted darunavir

**Fig 2:**
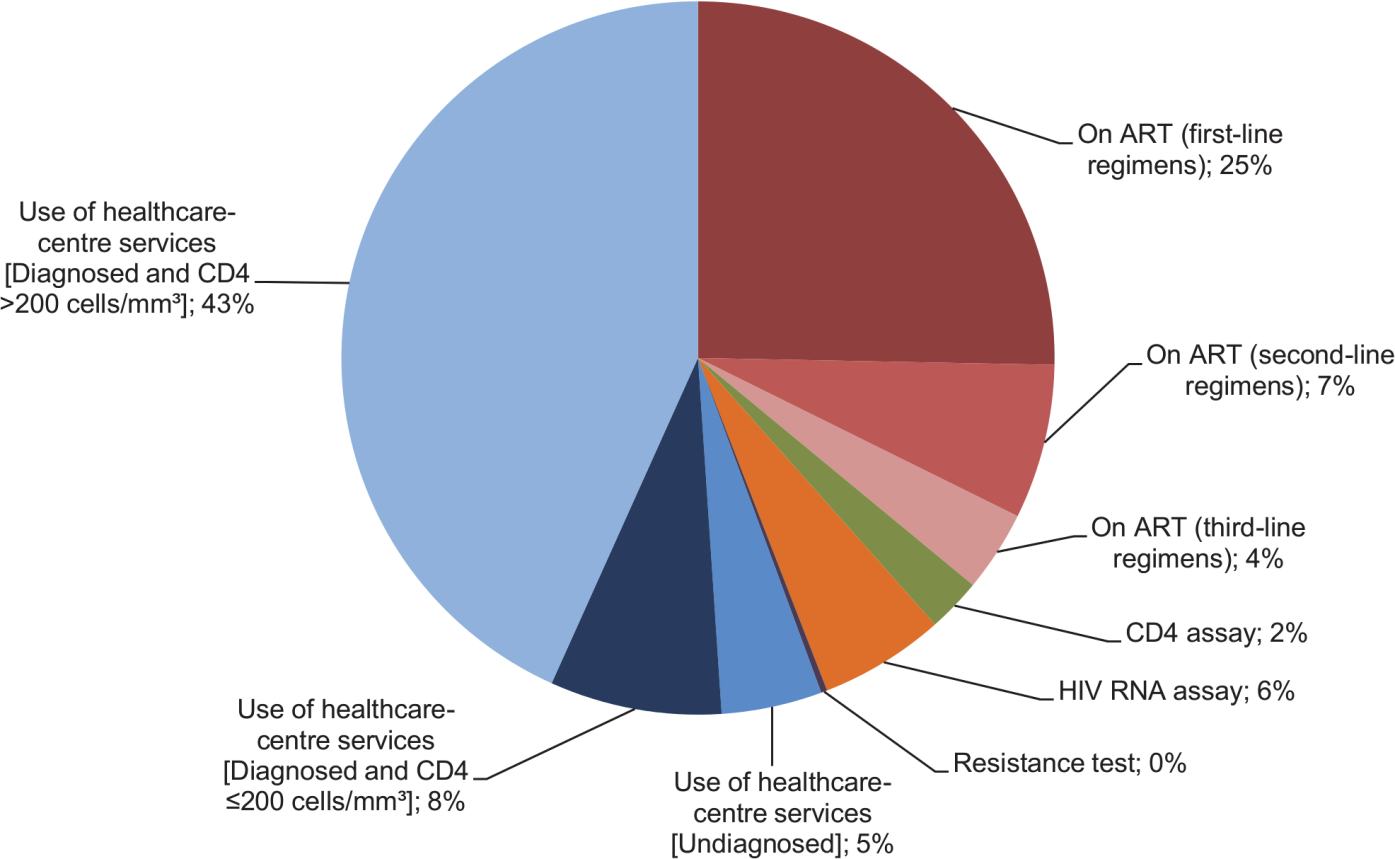
Distribution of costs spent in an average lifetime in analysis if patented drugs are replaced by generic versions.

Under the assumption that patented antiretroviral drugs are replaced by generics three years after patent expiry, the estimated mean lifetime cost was £179,600 and £101,200 discounted(**Table 3**). Therefore, by using generics with cost 20% that of patented drugs, the mean discounted lifetime costs were reduced by over £80,000 per person. In this scenario, costs of antiretrovirals contributed only 36% of the non-discounted projected lifetime costs, and use of healthcare centre services were now the largest cost burden(**Fig 2**). The average total undiscounted costs of first-line, second-line and third-line regimens were £45,500, £12,600 and £6,600 respectively.

If CD4 count measures were taken yearly (instead of 3-monthly) in individuals with suppressed viral load and previously high CD4 counts, the estimated mean lifetime cost was £357,500 (£183,800 discounted). CD4 count costs fell by £2,500 (a reduction of 57% compared to CD4 count costs in the base-case scenario). If virologically suppressed individuals with no history of virologic failure were switched from a cART regimen to DAR/r mono-therapy regimen, then the estimated mean lifetime cost was £330,600 (£169,200 discounted). Total ART costs fell by £30,400 (a reduction of 12% compared to ART costs in the base-case scenario). Reducing the frequency of healthcare centre visits to 6-monthly and annually in stably suppressed people (virologically suppressed, CD4 count >350 cells/mm^3^ at most recent visit and with no history of virologic failure) reduced mean lifetime costs to £344,000 and £334,900 respectively. Healthcare centre visits costs were accordingly reduced by 20% and 37%. All alternative strategies did not change the projected life expectancy by more than 15 months except for the switch to DAR/r mono-therapy, which resulted in a median life expectancy of 74.3 years (probably due to the modelled high resistance barrier and high potency of bPIs).

We performed univariable sensitivity analysis to assess several key assumptions which were made in the model (**Table 4**). If the assumed average age at infection is altered, it impacts both healthcare centre visit costs and ART costs due to the difference in number of years lived with HIV. If the rate of diagnosis is lowered such that MSM are only diagnosed when symptomatic or if AIDS develops, mean lifetime costs (and life expectancy) are reduced, mainly due to a 21% reduction in ART costs. Our results were not very sensitive to a slight change in the population distribution of adherence assumed in the model. However, if a much poorer distribution is assumed, this reduces ART costs by 38% as people with poor tendency to adhere are modelled to have a higher chance of treatment interruption and loss to care. The median lifetime cost from the multivariable sensitivity analysis was £328,300 and the 95% uncertainty bounds were (277,400–376,600). Further details are given in **S1 File**.

**Table 4:** Mean lifetime costs under different model assumptions (sensitivity analysis results).

Assumption in base-case analysis	New assumption	Mean lifetime costs (2013 £)	Discounted at 3.5% (2013 £)
Base-case analysis	-	360,800	185,200
Infected at age 30 years	Infected at age 20 years	432,400	201,500
	Infected at age 40 years	297,800	169,900
Rate of diagnosis in line with that currently observed (median CD4 count at diagnosis = 422 cells/mm^3^)	Diagnosed almost immediately after infection	371,000	194,300
	Diagnosed only when symptomatic or develop AIDS	294,000	148,700
Never lost from care	5% per year loss to care rate (return to care only when symptomatic or develop AIDS)	353,440	182,100
Initiate ART when CD4 count drops below 350 cells/mm^3^ (unless symptomatic)	Initiate ART when CD4 count drops below 500 cells/mm^3^ (unless symptomatic)	361,800	188,600
	Initiate ART soon after HIV diagnosis (unless symptomatic)	366,100	192,400
1.5-fold increased risk of non-AIDS deaths (compared to the general population)	1.1-fold increased risk of non-AIDS deaths (compared to the general population)	387,400	193,800
	1.25-fold increased risk of non-AIDS deaths (compared to the general population)	396,400	201,000
	1.5-fold increased risk of non-AIDS deaths but 2-fold in people with unsuppressed viral load (compared to the general population)	358,600	184,500
	1.5-fold increased risk of non-AIDS deaths (compared to the general population) and 1.5-fold increased healthcare centre visit costs whilst CD4 count <200 cells/mm^3^	404,500	208,500
Population distribution of adherence^a^ calibrated to data on proportion of men with suppressed viral load	Better population distribution of adherence	371,500	189,200
	Slightly worse population distribution of adherence	359,400	185,000
	Worse population distribution of adherence	241,300	140,200
Patented drugs replaced by generic versions (80% reduction in price) and population distribution of adherence^a^ calibrated to data on proportion of men with suppressed viral load	Patented drugs replaced by generic versions (80% reduction in price) and slightly worse population distribution of adherence	178,400	100,900
	Patented drugs replaced by generic versions (80% reduction in price) and worse population distribution of adherence	136,900	86,500
Healthcare centre visit costs incurred while undiagnosed are the same as those of someone who is diagnosed but with CD4 count >200 cells/mm^3^	No healthcare centre visit costs incurred while undiagnosed	348,300	176,100

ART: antiretroviral therapy

^**a**^ Further information on the modelled population distribution of adherence is explained in **S1 File**. In the base-case analysis, we use adherence pattern 2. Better population distribution refers to adherence pattern 1, slightly worse population distribution refers to adherence pattern 3 and worse population distribution refers to adherence pattern 5.

## Discussion

Based on the observed continuing low rates of virologic failure in treated individuals[21–23], predicted life expectancy in people with HIV is high in settings with access to good healthcare. Assuming that the current standards of care remain as they are, the mean lifetime healthcare cost of an MSM infected with HIV in 2013 at age 30 is estimated to be £360,800 (£185,200 discounted). If 3,000 MSM had been infected in 2013 (but diagnosed in later years) and all were aged 30 years at infection then the future direct lifetime costs relating to HIV care amounts to approximately £1.1 billion. Even with the future use of generics, the total sum remains in excess of £0.5 billion.

In the base-case scenario, we have made the assumption that healthcare centre costs (not including ART or HIV specific tests) of someone undiagnosed is the same as someone diagnosed with CD4 count >200 cells/mm^3^. The lifetime cost estimates may therefore have been overestimated because if someone is undiagnosed they would often not incur HIV-related care costs. However, this may be balanced out because we have not included costs of treating non-AIDS conditions which may be seen outside routine HIV care, such as GP appointments and emergency hospital admissions. As a result, modifying the costs incurred whilst someone is undiagnosed to £0 changes the lifetime cost estimates only slightly to £348,300.

We assumed that the efficacy and tolerability of generics were the same as patented drugs, though we vary the assumption of adherence in sensitivity analyses. Some studies have shown that single-pill regimens improve adherence and reduce hospitalisation[24, 25]. Nevertheless, future reductions in drug prices, due to a natural decline in prices and use of generics would reduce average lifetime costs considerably, given they form the largest part of HIV care costs. On the other hand, we continue to see improvements in life expectancy due to earlier diagnosis and advances in HIV treatment and care, which may mean that these cost reductions are somewhat attenuated due to the increased number of years in HIV care. In any case, our results provide updated information that can help to put the costs of potential HIV prevention interventions into perspective.

From a care provider prospective, it is plausible to reduce costs by way of reduced frequencies of CD4 count monitoring and clinic visits. However, our estimates of projected lifetime costs did not change substantially if CD4 count measures were taken yearly in individuals with suppressed viral load and CD4 count >350 cells/mm^3^. Reducing healthcare centre visit frequencies to 6-monthly or annually saved on average £15,000 to £25,000 per person in a lifetime. Although as a proportion of the estimated lifetime costs these savings may seem small, in healthcare centres with large numbers of people in care, the total costs saved may be substantial.

Conversely, costs relating to quantity of antiretroviral drugs are not as easy to reduce. The strategy in which all virologically-suppressed individuals with no history of virological failure were switched to DAR/r did actually reduce lifetime drug costs by 12%. However, given that individuals in the MONET and MONOI trials[26, 27] who were randomised to switch to the bPI mono-therapy arms (compared with staying on standard cART regimens) had a slightly higher risk of viral load rise, switching to bPI mono-therapy may only be suitable for those with nucleoside-related toxicities, rather than switching for cost-saving purposes.

Further cost reductions may be possible if these alternative strategies are combined with other cost-saving approaches such as cheaper point-of-care assays and using large purpose-built treatment centres. Note that our estimates of lifetime costs do not include non-HIV related healthcare costs, such as costs relating to the treatment of cardiovascular diseases and cancers, nor do they include loss of income associated with illness.

There have only been a few studies which have investigated lifetime costs of HIV in the recent cART-era. In 2006, Schackman et al.[28] estimated that from time of entry into HIV care, an adult starting treatment with CD4 count <350 cells/mm^3^ had a projected life expectancy of 24.2 years and projected lifetime cost of $618,900 in 2004 US dollars (approximately £500,000 in 2013 UK pounds). In 2012, Sloan et al.[29] provided an update of an earlier study[30] and projected a mean life expectancy of 26.5 years and lifetime cost of €535,000 in 2010 Euros (approximately £485,000 in 2013 UK pounds) for their simulated cohort with mean age 38 years who started cART with CD4 count <350 cells/mm^3^. Although it is not suitable to directly compare given the differences in study setting, it is still interesting to note that our estimates are considerably below either of these (albeit that our life expectancy estimate is higher), but all are consistent in that around 60 to 70% of costs are attributed to antiretroviral drug costs. Given that we have assumed an increased risk for non-AIDS deaths, when healthcare centre visit costs were also increased by 1.5-fold in diagnosed MSM with CD4 count <200 cells/mm^3^, the estimated lifetime healthcare costs rose to £404,500.

Walensky et al. have also recently assessed the cost-effectiveness of generics compared to patented drugs[31]. They found that a three-pill generic-based regimen reduced lifetime costs by $42,500 (approximately £27,000 in 2013 UK pounds). Survival decreased by 0.37 years however, as they assumed a small increase in rate of virological failure when on generic-based regimens.

Our analysis has a number of limitations. Firstly, due to modelling lifetime costs, we are inherently modelling over a long period of time. This means that the effect of discounting has a large impact. It also requires us to make an assumption that all-cause death rates remain at 2011 levels, rather than assuming they will continue on the current downward trajectory. It should also be noted that with an infectious disease such as HIV, evaluating the benefit of an intervention for reducing incidence of new infections must take into account reductions in future transmissions originating from the infected person[32]. Ideally this requires a dynamic mathematical model that includes uninfected people and accounts for transmission to HIV uninfected people from the concomitant infected population. This is beyond the scope of this paper, but should be borne in mind in any economic assessments of HIV prevention activities. Likewise, we did not account for quality of life. Such factors would need to be considered in a full economic evaluation.

In view of the high lifetime care costs for HIV-positive individuals, there is large scope for preventative interventions to be cost-effective. Our results show that for settings with good access to cART and HIV care, it is imperative for investment into prevention programmes to be continued or scaled-up. Future reductions in drug prices by using generic antiretroviral drugs in place of patented drugs would reduce these costs considerably.

## Supporting Information

S1 File Projected lifetime healthcare costs associated with HIV infection. Supplementary Material. January 2015.
(DOCX)
