# Supplementary material for: Projected Lifetime Healthcare Costs Associated with HIV Infection
Source: PLoS One. 2015 Apr 22;10(4):e0125018. doi: 10.1371/journal.pone.0125018 (PMC4406522; doi:10.1371/journal.pone.0125018)
Supplement: S1 File — Supplementary Material. January 2015. (DOCX) [file pone.0125018.s001.docx]

Projected lifetime healthcare costs associated with HIV infection

Supplementary Material

January 2015

Contents

[1 Brief description of HIV Synthesis Progression model 5](#_Toc415150260)

[2 Analysis details 6](#_Toc415150261)

[2.1 Modifications made for this manuscript 6](#_Toc415150262)

[2.2 Determination of date of diagnosis 6](#_Toc415150263)

[3 Full model details 7](#_Toc415150264)

[3.1 Natural history 7](#_Toc415150265)

[3.1.1 Parameter values and distributions 7](#_Toc415150266)

[3.1.2 Viral load 7](#_Toc415150267)

[3.1.3 CD4 count 7](#_Toc415150268)

[3.1.4 X4 virus 8](#_Toc415150269)

[3.2 Use of ART 8](#_Toc415150270)

[3.2.1 Parameter values and distributions 8](#_Toc415150271)

[3.2.2 Initiation of ART 9](#_Toc415150272)

[3.2.3 Antiretroviral drugs 9](#_Toc415150273)

[3.2.4 Interruption of ART 9](#_Toc415150274)

[3.2.5 Interruption of ART without clinic/clinician being aware 9](#_Toc415150275)

[3.2.6 Adherence 10](#_Toc415150276)

[3.3 Effect of ART on viral load, CD4 count and resistance development 11](#_Toc415150277)

[3.3.1 Parameter values and distributions 11](#_Toc415150278)

[3.3.2 Determination of viral load, CD4 count, resistance development whilst on ART 11](#_Toc415150279)

[3.3.3 Viral load (mean change from viral load max), CD4 count change (mean change between t-1 and t), and new mutation risk in first 3 months 13](#_Toc415150280)

[3.3.4 Summary of viral load between 3-6 months since starting current regimen and after 6 months if viral load at t-1 > 4 log copies/ml 14](#_Toc415150281)

[3.3.5 Summary of CD4 count change (mean change between t-1 and t) between 3-6 months since starting current regimen and after 6 months if viral load at t-1 > 4 log copies/ml 15](#_Toc415150282)

[3.3.6 Summary of new mutation risk between 3-6 months, and after 6 months if viral load at t-1 > 4 log copies/ml 16](#_Toc415150283)

[3.3.7 Summary of viral load (mean change from viral load max), CD4 count change (mean change between t-1 and t), after 6 months, where viral load at t-1 < 4 log copies/ml. 17](#_Toc415150284)

[3.3.8 Changes in viral load, CD4 count and new mutation risk if the number of active drugs in current regimen = 0 18](#_Toc415150285)

[3.3.9 Factors which affect the CD4 count rise 18](#_Toc415150286)

[3.3.10 Viral load and CD4 count changes during ART interruption 19](#_Toc415150287)

[3.4 Resistance 19](#_Toc415150288)

[3.4.1 Modelling resistance 19](#_Toc415150289)

[3.4.2 Accumulation of resistance mutations 20](#_Toc415150290)

[3.4.3 Loss of acquired mutations from majority virus 21](#_Toc415150291)

[3.4.4 “Regaining” mutations in majority virus after restarting ART 22](#_Toc415150292)

[3.4.5 Determination of level of resistance to each drug 22](#_Toc415150293)

[3.4.6 Calculation of activity level of drug 24](#_Toc415150294)

[3.5 Toxicity 24](#_Toc415150295)

[3.5.1 Incidence of new current toxicity 24](#_Toc415150296)

[3.5.2 Switching of drugs due to toxicity 25](#_Toc415150297)

[3.6 Risk of clinical disease and death 25](#_Toc415150298)

[3.6.1 Parameter values and distributions 26](#_Toc415150299)

[3.6.2 Occurrence of AIDS 26](#_Toc415150300)

[3.6.3 Occurrence of WHO 3 diseases 27](#_Toc415150301)

[3.6.4 Occurrence of HIV-related deaths 27](#_Toc415150302)

[3.6.5 Occurrence of non-HIV-related deaths 27](#_Toc415150303)

[4 Model fits 28](#_Toc415150304)

[4.1 Incubation period to AIDS and death from seroconversion (no ART) 28](#_Toc415150305)

[4.1.1 Incubation period to AIDS (no ART) stratified by sex and race (black vs white). Observed data from reference [13]. 28](#_Toc415150306)

[4.1.2 Incubation period to AIDS (no ART) stratified by age. Dotted line shows modelled data. Observed data from reference [13]. 28](#_Toc415150307)

[4.1.3 Incubation period to death (no ART) stratified by sex and race (black vs white). Observed data from reference [13]. 29](#_Toc415150308)

[4.1.4 Incubation period from AIDS to death (no ART). Observed data from reference [73]. 29](#_Toc415150309)

[4.1.5 Time to CD4 count <200, <350, <500 cells/mm^3^ (no ART). Observed data from reference [84]. 30](#_Toc415150310)

[4.2 Other model fits relating to the natural history of HIV 30](#_Toc415150311)

[4.2.1 Viral load set point and initial CD4 count (after primary infection). Observed data from reference [85] 30](#_Toc415150312)

[4.2.2 Association between viral load measured close to seroconversion (between 6-24 months) and risk of AIDS, adjusting for CD4 count and age. Observed data from reference [8]. 30](#_Toc415150313)

[4.2.3 Cumulative 6-year risk of AIDS by CD4 count and viral load and age in the absence of ART. Observed data from reference [12]. 31](#_Toc415150314)

[4.2.4 Median CD4 count at diagnosis of AIDS and at death (No ART). Observed data from reference [71] 31](#_Toc415150315)

[4.3 Model fits relating to the effect of ART 32](#_Toc415150316)

[4.3.1 3 year percent risk of AIDS after start of ART by baseline CD4 / viral load (age < 50, non-IDU, AIDS-free). Observed data from reference [86]. 32](#_Toc415150317)

[4.3.2 % with virologic failure (viral load > 500 copies/mL / on ART) by time from start of HAART (patients starting with PI/r or NNRTI regimen). Observed data from reference [87]. 32](#_Toc415150318)

[4.3.3 Effect of HAART vs. no therapy on risk of AIDS and death. Observed data from reference [88]. 33](#_Toc415150319)

[4.3.4 Rate of viral rebound in people on 1st line HAART and with viral load < 50 copies/mL. Observed data from reference [89]. 33](#_Toc415150320)

[4.3.5 Median CD4 count change at 3 years from start of HAART. Observed data from reference [38]. 33](#_Toc415150321)

[4.3.6 Discontinuation of drugs in initial HAART regimen. Observed data from reference [90]. 33](#_Toc415150322)

[4.3.7 Percent with triple class virologic failure by years from start of HAART (patients naïve before HAART). Observed data from reference [91]. 34](#_Toc415150323)

[4.3.8 Triple class failure (those with triple class failure before 2001). Observed data from reference [47]. 34](#_Toc415150324)

[4.3.9 Percent with triple class virological failure by years from start of HAART (patients naïve before HAART). Observed data from reference [92]. 34](#_Toc415150325)

[4.3.10 Risk of death after triple class virologic failure. Observed data from reference [47]. 35](#_Toc415150326)

[4.4 Model fits relating to resistance 35](#_Toc415150327)

[4.4.1 Risk of resistance mutations (and virologic failure) after start of ART (patients starting with PI/r or NNRTI regimen). Observed data from reference [87]. 35](#_Toc415150328)

[4.4.2 % with at least one resistance mutation for all three main classes (and virologic failure). Observed data from reference [39]. 35](#_Toc415150329)

[4.4.3 Risk of resistance mutations after start of ART. Observed data from reference [39]. 36](#_Toc415150330)

[4.4.4 Risk of death after triple class resistance. Observed data from reference [93]. 36](#_Toc415150331)

[5 Sensitivity analyses 37](#_Toc415150332)

# Brief description of HIV Synthesis Progression model

The HIV Synthesis Progression model is an individual-based stochastic computer simulation model of HIV progression and the treatment of HIV infection. The model was originally developed by Phillips and colleagues to reconstruct the HIV-infected population in the UK[1]. It incorporates our understanding of the underlying processes of HIV disease progression and the effect of ART, based on data from clinical trials and epidemiological data.

The model has been recently updated; the current version is Synthesis V6. Synthesis V5 was updated to V6 based on a complete re-evaluation of every parameter value and also some small additions to the model structure.

In brief, the Synthesis Progression model generates simulated ‘data’ on the progression of HIV infection and effect of ART on simulated patients. Each patient in the model is simulated from the time of infection (although for simplicity we do not explicitly model acute changes in viral load and CD4 count around the time of seroconversion) and they are followed until either death, loss to emigration or to any given calendar year of interest. For each simulated person, the model generates variables such as calendar date, CD4 cell count, viral load, age, presence of transmitted resistance mutations. The values of these variables are updated every three months in the model. Use of specific antiretroviral drugs, adherence, accumulation of resistance mutations and clinical events are also modelled in order to incorporate the effect of ART. The progression model has been shown to provide a generally close fit to observed data relating to the natural progression and therapy outcomes[1-3].

# Analysis details

## Modifications made for this manuscript

The HIV Synthesis progression model was originally developed to reconstruct the HIV-infected population in the UK and to predict future trends in key outcomes. For the purposes of this paper, the model was modified in the following ways:

- All simulated people assumed to be MSM, assumed to be living in the UK at HIV infection
- All MSM are infected with drug-sensitive virus in 2013, aged 30 and outcomes are simulated until 2093 or until death (whichever occurs earlier)
- All MSM are assumed never to be lost to follow-up or emigrate throughout their lifetime

We simulated a population of 10,000 MSM as described above. The fitting was by subjective judgement informed by knowledge of the data sources, but not by a formal measurement of goodness of fit. By showing the fit of the model to a wide range of diverse data sources relevant to different parameters (see section 4), we consider to have demonstrated that we have a reasonably well fitting model; readers (including non-technical readers) can judge for themselves the adequacy of the fit. We acknowledge, however, that the fact that we have not arrived at parameter values through some formal and/or automated fitting procedure is a limitation and we cannot rule out that there are parameter value combinations that would give a better fit.

## Determination of date of diagnosis

The probability of being diagnosed with HIV in a 3 month period is 0.05. This was chosen to reflect what has been observed recently in the UK for MSM in terms of CD4 count at diagnosis, i.e. median CD4 count of 422 cells/mm^3^ and 35% diagnosed late (CD4 count <350 cells/mm^3^ within 3 months of diagnosis) in 2011[4]. The diagnosis rate is further determined by a number of factors. HIV will be definitely be diagnosed if AIDS occurs. If CDC B symptoms occur there is a 50% probability that HIV is diagnosed at that point. Subsequently, if CDC B symptoms have occurred there is a 5-fold increased probability of diagnosis. Patients who have a general tendency to be non-adherent to care (and to ART if and when they start ART), have a 2-fold reduced rate of diagnosis compared with the usual rate of diagnosis (see section 3.2.6 for more on adherence).

The figure below shows the rate of diagnosis in our simulated population of 10,000 MSM infected in 2013:

If the person acquired HIV is an MSM, then there is a 10% probability of being diagnosed during primary infection, which is only when $t=1$.

# Full model details

Here we describe the details of the model. For each variable we outline how it is generated and what are the factors on which it depends. Variables are updated in 3 month intervals, i.e. period *t-1* to *t* and period *t* to *t+1* are both 3 month time intervals.

## Natural history

These estimates are derived based on synthesis of evidence from natural history studies[5-12] and were selected in conjunction with other relevant parameter values to provide a good fit to the incubation period distribution. Viral subtype is currently not specified; data to which it is fitted are mainly from Europe and so will reflect the subtypes in circulation, i.e. mainly subtype B.

### Parameter values and distributions

For HIV infected people the variables modelled include: primary infection (a period of raised infectivity of 3 months duration), viral load, CD4 cell count, presence of specific resistance mutations, adherence to ART, risk of AIDS and death. The model of progression of HIV and the effect of ART has been shown to provide a generally close fit to observed data relating to natural progression of HIV infection, comparing the output of the model with data coming mainly from observational studies conducted in Europe for the natural history (incubation period)[1, 13, 14].

| **Parameter** | | **Value (or distribution) where applicable** |
| --- | --- | --- |
| **Variable name in program** | **Description** |  |
| $v\{t\}$ | Viral load, log_10_ scale | $Min\left( v\left\{ t \right\} \right)=0$ $Max\left( v\left\{ t \right\} \right)=6.5$ |
| $V_{set}$ | Viral load at set point | $Normal\left( 4,{0.5}^{2} \right)$ |
| $vc\{t-1\}$ | Change in viral load from ($t-1)$ to $t$ |  |
| *gx* | Factor adjusting basic rate of natural viral load change | 1.0 |
| $c\{t\}$ | CD4 count, square root scale | $Min\left( c\left\{ t \right\} \right)=0$ |
| $ccsqr\{t-1\}$ | Change in CD4 count from ($t-1)$ to $t$, square root scale |  |
| *mean_sqrtcd4_inf* | Initial CD4 count at infection, square root scale | $Normal(29.5,2^{2})$ |
| *fx* | Factor adjusting basic rate of natural CD4 count decline | 1.0 |
| *sd_cd4* | Standard deviation of CD4 count change | $Normal(1.2,0.2^{2})$ |

### Viral load

The initial log_10_ viral load, $v\{1\}$, is assumed to be the viral load reached after primary infection. For any person, it is given by:

$$v\left\{ 1 \right\}=V_{set}+\left[ \left( age\left\{ 1 \right\}-35 \right)\times0.005 \right]-\left[ 0.1 \text{if female} \right]$$

Viral load change from period ($t-1)$ to $t$, $vc\{t-1\}$, is given by

$$vc\left\{ t-1 \right\}=gx\times0.0225+ \left[ \left( age\left\{ t-1 \right\}-35 \right)\times0.0005 \right]+Normal\left( 0,{0.05}^{2} \right)$$

### CD4 count

Initial CD4 count, modelled on the square root scale, *c{1}*, is dependent on *V_set_*, *age{1}* and race and is given by

$$c\left\{ 1 \right\}=mean\_sqrtcd4\_inf- \left( 1.5\times V_{set} \right)+\left[ \left( age\left\{ 1 \right\}-35 \right)\times0.05 \right]-[2 \text{if black race}]$$

$$\text{where }min\left( c\left\{ 1 \right\} \right)=18 \text{and }\max\left( c\left\{ 1 \right\} \right)=38.7$$

As for viral load, no attempt is made to model the dynamic CD4 count changes in primary infection – the viral load and the CD4 count are both assumed to have reached its settled state right from the first period.

The change in CD4 count from period ($t-1)$ to $t$, *ccsqr{t-1}*, are dependent on the current viral load (i.e. viral load at time t-1, *v{t-1}*) and are given by sampling from a Normal distribution with mean *fx* and variance *(sd_cd4)^2^* multiplied by the values as follows:

| **Viral load at t-1, *v{t-1}*** | **Mean square root CD4 change (per 3 months), *ccsqr{t-1}*** |
| --- | --- |
| < 2.5 | 0 |
| 2.5- | -0.05 |
| 3.0- | -0.15 |
| 3.5- | -0.25 |
| 4.0- | -0.30 |
| 4.5- | -0.45 |
| 5.0- | -0.95 |
| 5.5- | -1.55 |
| 6.0- | -1.75 |

The CD4 count change, $ccsqr\{t-1\}$, from period ($t-1)$ to $t$ is further dependent on race (0.05 less decline if black race) and of X4-tropic virus (0.25 further decline if X4-tropic).

### X4 virus

Initial virus is assumed to be R5-tropic. Shift to presence of X4 virus is assumed to depend on viral load. The probability of shift in a 3-month period is given by, $pr\_x4\_shift$, which is distributed as follows:

This translates into a rate of 5% per year in a person with viral load 30,000 copies/ml and 16% per year in a person with 100,000 copies/ml, which are broadly consistent with observed data[15].

## Use of ART

### Parameter values and distributions

| **Parameter** | | **Value (or distribution) where applicable** |
| --- | --- | --- |
| **Variable name in program** | **Description** |  |
| *Prob_art* | Probability of initiating ART when eligible per 3 months | 0.8 |
| *will_take_enf* | Willingness to take enfuvirtide | 0.85 |
| *rate_inter* | Probability of interruption per 3 months | $0.01$ |
| *clinic_not_aware{t}* | If patient interrupting treatment, whether the clinic(ian) is aware or not |  |
| *clinic_not_aware_frac* | Proportion of interruptions where clinic/clinician is not aware of the interruption | 0.3 |
| *rate_restart* | Probability of restarting following interruption per 3 months | 0.6 |
| *adh{t}* | Adherence | 0≤adh{t}≤1 |
| *adhav* | Adherence average, fixed value for each person | 0≤adhav≤1 |
| *adhvar* | Period-to-period variability of adherence average | 0.05≤adhvar≤0.2 |
| *e_adh{t}* | Effective adherence | 0≤e_adh{t}≤1 |

### Initiation of ART

ART initiation in diagnosed people is determined by a CD4 count <350 cells/mm^3^ or the development of AIDS. The probability of initiating per 3 months is given by *prob_art*. If the person presents with symptoms or AIDS, regardless of CD4 count, then the probability of initiating per 3 months is 1.1-fold and 1.25-fold higher respectively.

*prob_art* has been informed by recommendations and guidelines of when to start ART in Europe[16-18].

### Antiretroviral drugs

All antiretroviral drugs are modelled separately. Drugs (abbreviations used throughout this documentation) modelled are: zidovudine (ZDV), stavudine (D4T), didanosine (DDI), lamivudine (3TC), abacavir (ABA), emtricitabine (FTC), tenofovir (TEN), new nucleosides (NNU), nevirapine (NEV), efavirenz (EFA), etravirine (ETR), saquinavir (SAQ), ritonavir (RIT), indinavir (IND), nelfinavir (NEL), lopinavir/r (LPR), amprenavir/r (AMP), atazanavir/r (TAZ), darunavir (DAR), maraviroc (MAR), raltegravir (RAL) and enfuvirtide (ENF).

There is evidence that not all patients are willing to take enfuvirtide[19]. We assume that a proportion, *will_take_enf*, of patients who are willing to take it.

### Interruption of ART

All interruptions are assumed to be patient choice, as opposed to drug supply shortage.

The basic rate of interruption, *rate_inter*, is greater with current toxicity (2-fold) and greater in patients with a greater tendency to be non-adherent (1.5-fold if adherence average 0.5-0.79 and 2-fold if adherence average <0.50). Younger people (0.11-fold increase per year older) and people currently with lactic acidosis (100-fold) also have a higher probability of interruption.

The rate of interruption is likely to vary by setting. The above rates were derived to be consistent with data from mainly European and US cohorts[20-23].

### Interruption of ART without clinic/clinician being aware

It is known that in some instances, people on ART have poor adherence that they have in fact interrupted or stopped ART entirely but, in the same way that the clinician is not always aware of the true adherence level, they are also not always aware when the person has completely interrupted ART. This means that the clinician may think a patient is virologically failing, because viral load is high, when in fact this is due to interruption rather than resistance. This can be seen from studies on people with virologic failure in which a proportion have no identified resistance mutations[24, 25]. Thus, when a person interrupts ART (but remains under care) we introduce a variable that indicates whether the clinician is unaware, *clinic_not_aware{t}*. The proportion of people who have interrupted, but where the clinic/clinician is not aware, is given by *clinic_not_aware_frac*. If a patient has interrupted ART with the clinician unaware then not only is the patient (wrongly) classified (by the clinician) as virologically failing, but a switch to second/third line can occur.

### Adherence

There are two components to the adherence. Each patient has a fixed “tendency to adhere” but their actual adherence varies from period to period, both at random and according to the presence of symptoms. Adherence is measured on a scale of 0 to 1.

#### Component which is fixed over time for a given patient

Adherence average, *adhav*, is a measure of the patient’s tendency to adhere, a fixed value for a patient, with a certain period-to-period variability, *adhvar*. Adherence at any one period is determined as follows (although with modifications explained below):

$$adh\left\{ t \right\}=adhav+Normal(0,adhvar)$$

$$\text{where }min\left( adh\left\{ t \right\} \right)=0 \text{and }\max\left( adh\left\{ t \right\} \right)=1$$

There are various adherence pattern distributions (numbered 1-5) considered:

| **Adherence pattern** | **Probability** | ***adhav*** | ***adhvar*** |
| --- | --- | --- | --- |
| 1 | 3% | 0.5 | 0.2 |
|  | 3% | 0.8 | 0.2 |
|  | 14% | 0.9 | 0.06 |
|  | 80% | 0.95 | 0.05 |
| 2 | 5% | 0.5 | 0.2 |
|  | 10% | 0.8 | 0.2 |
|  | 27% | 0.9 | 0.06 |
|  | 38% | 0.9 | 0.05 |
|  | 20% | 0.95 | 0.05 |
| 3 | 15% | 0.5 | 0.2 |
|  | 15% | 0.7 | 0.2 |
|  | 50% | 0.9 | 0.06 |
|  | 20% | 0.95 | 0.05 |
| 4 | 30% | 0.5 | 0.2 |
|  | 30% | 0.7 | 0.2 |
|  | 10% | 0.9 | 0.06 |
|  | 30% | 0.95 | 0.05 |
| 5 | 30% | 0.5 | 0.2 |
|  | 30% | 0.6 | 0.2 |
|  | 10% | 0.7 | 0.06 |
|  | 30% | 0.9 | 0.05 |

In the base-case analysis, we use adherence pattern 2. These estimates are based partially on observed adherence data[26-31], but also on adherence levels required to produce observed estimates of rates of resistance development and virologic failure (see model fit below) and also data on the proportion of patients at first virologic failure who have no resistance mutations present[32]. It is clear from such data in more recent years that the great majority of patients who started ART with three or more drugs are sufficiently adherent that virologic failure rates are low (and so resistance accumulation is also likely to have been slow)[33, 34].

#### Higher adherence associated with older age

Rates of viral suppression, which is often a surrogate measure for adherence, are higher in people who are older[35]. For every 1 year increase in age, *adh{t}* is higher by 0.002. The ages for which this effect holds is limited to between 16 and 70.

#### Effective adherence

We also considered the concept of effective adherence, *e_adh{t}*, which reflects predicted adequacy of drug levels.

It is assumed that patients on ART are susceptible to occasional severe temporary drops in drug level (i.e. level of *e_adh{t}*) at a rate of 0.02 events per year. This leaves them susceptible to viral rebound, but with low risk of resistance as the effective adherence drop is so profound. This phenomenon is assumed to be 3 times more frequent among those on protease inhibitor regimens. This latter assumption is the only plausible means (at least within our model framework) to explain why virologic failure occurring on boosted protease inhibitor regimens often occurs in the absence of resistance.

If a patient’s (current) measured CD4 count is less than 200 cells/mm^3^ and they have also had triple-class failure in the past (where triple-class failure is defined as virological failure of at least two NRTIs, one NNRTI and a boosted-PI), then the patient’s effective adherence can also increase by an additive factor of 0.25. The rationale for this is that at some point it is assumed that when a person is facing ultimate failure of ART and clinical progression, they will be particularly motivated to adhere to ART.

## Effect of ART on viral load, CD4 count and resistance development

### Parameter values and distributions

| **Parameter** | | **Value (or distribution) where applicable** |
| --- | --- | --- |
| **Variable name in program** | **Description** |  |
| *newmut{t}* | Probability of acquiring new resistance mutations |  |
| *nactive{t}* | Number of active drugs |  |
| *vmax* | Maximum ever viral load, log_10_ scale |  |
| *pt_cd4_rise_art* | Propensity for CD4 count rise whilst on ART, fixed for each person | $e^{0.2}$ |
| *cmax* | Maximum CD4 count to which can return on ART | $e^{6.6}$ |
| *poorer_cd4_rise_on_failing_nnrti* | Extent to which CD4 count change is more favourable on a virologically failing bPI-regimen compared with an NNRTI-regimen | $-6$ |
| *cmin{t}* | CD4 count nadir, square root scale |  |

### Determination of viral load, CD4 count, resistance development whilst on ART

Potent ART regimens are known to reduce viral load, which in turn leads to recovery of CD4 cell counts[34, 36, 37]. Changes in the viral load and CD4 counts whilst an individual is on ART are modelled differently to when an individual is ART-naïve.

Determination of viral load, CD4 count, acquisition of new resistance mutations (variable *newmut{t}*) between ($t-1)$ to $t$ depend on: effective adherence between ($t-1)$ to $t$, number of active drugs (*nactive{t-1}*)), time on the current regimen and the current viral load itself. The way the values are generated is detailed on the following pages. For those on NNRTI regimens the new mutations risk is assumed to be that for the effective adherence category of 0.5 – 0.8 (i.e. maximal) even if *e_adh{t} < 0.5*, reflecting the fact that NNRTI resistance develops easily, even when drug exposure is very low.

In the following sections, ‘starting current regimen’ means starting treatment for the first time as well as any treatment regimen following a treatment interruption.

The changes in viral load and CD4 count are based on observed data and observational studies (and to some extent randomized trials, although responses tend to be better in trial participants), and provide long term estimates of virologic failure rates and CD4 count increases in ART which are broadly consistent with observed. Values of the “new mutation risk” parameter, *newmut{t}*, have been chosen in conjunction with the translation of presence of mutations into reduce drug activity to provide estimates of resistance accumulation consistent with those observed in clinical practice[36-43].

### Viral load (mean change from viral load max), CD4 count change (mean change between t-1 and t), and new mutation risk in first 3 months

For 0 active drugs, these are the changes regardless of time from start of regimen.

The initial 3-month change in viral load is described as the mean change from the patient’s maximum viral load to that point (*vmax*) on the log scale. This is the mean of a normal distribution with variance 0.2^2^, from which the patient’s value/change is sampled.

The change in CD4 count is described as the mean change between periods ($t-1)$ to $t$. This change is then multiplied by a factor which represents each individual’s underlying propensity for CD4 count rise whilst on ART (given by *pt_cd4_rise_art*). If the mean CD4 count change obtained from the table below is positive, then the mean value is subsequently multiplied by this factor. However, if the CD4 count change in the table is a negative value (i.e. not a CD4 count rise), then it is not multiplied by this factor.

For the new mutation risk, this is a number that is multiplied by the viral load (mean of values at ($t-1)$ to $t$). The resulting number, *newmut{t}* is used when assessing whether a new mutation or mutations have arisen (see section 3.4.1).

|  |  | **Number of active drugs** | | | | | | | | | | | |
| --- | --- | --- | --- | --- | --- | --- | --- | --- | --- | --- | --- | --- | --- |
|  | **‘Effective**  **adherence’ between *t-1* & *t*** | **3** | **2.75** | **2.5** | **2.25** | **2** | **1.75** | **1.5** | **1.25** | **1** | **0.75** | **0.5** | **0.25** |
| **Viral load (log change from *vmax*)** | > 0.8 | -3 | -2.6 | -2.2 | -1.8 | -1.5 | -1.25 | -0.9 | -0.8 | -0.7 | -0.55 | -0.4 | -0.3 |
|  | > 0.5, < 0.8 | -2 | -1.6 | -1.2 | -1.1 | -0.9 | -0.8 | -0.6 | -0.5 | -0.4 | -0.25 | -0.1 | -0.05 |
|  | < 0.5 | -0.5 | -0.4 | -0.3 | -0.25 | -0.2 | -0.15 | 0 | 0.05 | 0.1 | 0.1 | 0.1 | 0.1 |
|  |  |  |  |  |  |  |  |  |  |  |  |  |  |
| **CD4 count change (*t-1* to *t*)** | > 0.8 | 70 | 45 | 40 | 35 | 30 | 25 | 20 | 17 | 13 | 10 | 5 | -2 |
|  | > 0.5, < 0.8 | 30 | 30 | 23 | 20 | 15 | 13 | 10 | 8 | 5 | 3 | 0 | -7 |
|  | < 0.5 | 5 | 4 | 3 | 2 | 1 | -1 | -3 | -6 | -10 | -11 | -12 | -13 |
|  |  |  |  |  |  |  |  |  |  |  |  |  |  |
| **New mutation risk (x log viral load)** | > 0.8 | 0.002 | 0.01 | 0.03 | 0.05 | 0.1 | 0.15 | 0.2 | 0.3 | 0.4 | 0.45 | 0.5 | 0.5 |
|  | > 0.5, < 0.8 | 0.15 | 0.15 | 0.2 | 0.25 | 0.3 | 0.3 | 0.3 | 0.35 | 0.4 | 0.45 | 0.5 | 0.5 |
|  | < 0.5 | 0.05 | 0.05 | 0.05 | 0.05 | 0.05 | 0.05 | 0.05 | 0.05 | 0.05 | 0.05 | 0.05 | 0.05 |

### Summary of viral load between 3-6 months since starting current regimen and after 6 months if viral load at t-1 > 4 log copies/ml

This table applies to patients for whom it has been between 3 and 6 months since starting their current regimen, as well as patients who have been on their current regimen for more than 6 months but who have a viral load > 4 log copies/ml (e.g. due to previous poor adherence). The change in viral load is described as the mean change from the patient’s maximum viral load to that point (*vmax*) on the log scale. Otherwise, if the number in the table is underlined, it is the mean absolute value. This is the mean of a normal distribution with variance 0.2^2^, from which the patient’s value/change is sampled.

|  |  | **Number of active drugs** | | | | | | | | | | | |
| --- | --- | --- | --- | --- | --- | --- | --- | --- | --- | --- | --- | --- | --- |
| **‘Effective adherence’ between *t-2* & *t-1*** | **‘Effective adherence’ between *t-1* & *t*** | **3** | **2.75** | **2.5** | **2.25** | **2** | **1.75** | **1.5** | **1.25** | **1** | **0.75** | **0.5** | **0.25** |
| > 0.8 | > 0.8 | 0.5 | 0.8 | 1.2 | 1.4 | 2.0 | 2.7 | -1.7 | -1.15 | -0.9 | -0.75 | -0.6 | -0.4 |
| > 0.5, < 0.8 | > 0.8 | 1.2 | 1.2 | 1.2 | 1.4 | -2.0 | -1.6 | -1.2 | -1.05 | -0.9 | -0.7 | -0.5 | -0.35 |
| < 0.5 | > 0.8 | 1.2 | 1.2 | 1.2 | 1.4 | -2.0 | -1.6 | -1.2 | -1.0 | -0.9 | -0.7 | -0.5 | -0.2 |
|  |  |  |  |  |  |  |  |  |  |  |  |  |  |
| > 0.8 | > 0.5, < 0.8 | 1.2 | 1.6 | 1.8 | 2.2 | 2.4 | -2.4 | -1.5 | -0.9 | -0.7 | -0.55 | -0.4 | -0.3 |
| > 0.5, < 0.8 | > 0.5, < 0.8 | 2.5 | 2.5 | 2.5 | 2.5 | -1.2 | -1.1 | -0.8 | -0.65 | -0.5 | -0.35 | -0.2 | -0.05 |
| < 0.5 | > 0.5, < 0.8 | -2.0 | -1.8 | -1.5 | -1.35 | -1.2 | -1.1 | -0.8 | -0.65 | -0.5 | -0.2 | -0.2 | -0.05 |
|  |  |  |  |  |  |  |  |  |  |  |  |  |  |
| > 0.8 | < 0.5 | -0.5 | -0.4 | -0.3 | -0.25 | -0.2 | -0.15 | -0.10 | -0.05 | +0 | +0 | +0 | +0 |
| > 0.5, < 0.8 | < 0.5 | -0.5 | -0.4 | -0.3 | -0.25 | -0.2 | -0.15 | -0.10 | -0.05 | +0 | +0 | +0 | +0 |
| < 0.5 | < 0.5 | -0.5 | -0.4 | -0.3 | -0.25 | -0.2 | -0.15 | -0.10 | -0.05 | +0 | +0 | +0 | +0 |

### Summary of CD4 count change (mean change between t-1 and t) between 3-6 months since starting current regimen and after 6 months if viral load at t-1 > 4 log copies/ml

This table applies to patients for whom it has been between 3 and 6 months since starting their current regimen, as well as patients who have been on their current regimen for more than 6 months but who have a viral load > 4 log/copies/ml (e.g. due to previous poor adherence).

The change in CD4 count is described as the mean change between periods ($t-1)$ to $t$. This change is then multiplied by a factor which represents each individual’s underlying propensity for CD4 count rise whilst on ART (given by *pt_cd4_rise_art*). If the mean CD4 count change obtained from the table below is positive, then the mean value is subsequently multiplied by this factor. However, if the CD4 count change in the table is a negative value (i.e. not a CD4 count rise), then it is not multiplied by this factor.

|  |  | **Number of active drugs** | | | | | | | | | | | |
| --- | --- | --- | --- | --- | --- | --- | --- | --- | --- | --- | --- | --- | --- |
| **‘Effective adherence’ between *t-2* & *t-1*** | **‘Effective adherence’ between *t-1* & *t*** | **3** | **2.75** | **2.5** | **2.25** | **2** | **1.75** | **1.5** | **1.25** | **1** | **0.75** | **0.5** | **0.25** |
| > 0.8 | > 0.8 | +30 | +28 | +25 | +23 | +21 | +19 | +3 | -5 | -9 | -10.5 | -12 | -14 |
| > 0.5, < 0.8 | > 0.8 | +30 | +28 | +25 | +23 | +7.5 | +1.5 | -4.5 | -7 | -9 | -11 | -13 | -14.5 |
| < 0.5 | > 0.8 | +30 | +28 | +25 | +23 | +7.5 | +1.5 | -4.5 | -7.5 | -9 | -11 | -13 | -16 |
|  |  |  |  |  |  |  |  |  |  |  |  |  |  |
| > 0.8 | > 0.5, < 0.8 | +15 | +13 | +10 | +8 | +7 | +4 | +0 | -9 | -11 | -12.5 | -14 | -15 |
| > 0.5, < 0.8 | > 0.5, < 0.8 | +15 | +13 | +10 | +8 | -4.5 | -6 | -10 | -11.5 | -13 | -14.5 | -16 | -17.5 |
| < 0.5 | > 0.5, < 0.8 | +7.5 | +4.5 | +0 | -2 | -4.5 | -6 | -10 | -11.5 | -13 | -16 | -16 | -17.5 |
|  |  |  |  |  |  |  |  |  |  |  |  |  |  |
| > 0.8 | < 0.5 | -13 | -14 | -15 | -15.5 | -16 | -16.5 | -17 | -17.5 | -18 | -18 | -18 | -18 |
| > 0.5, < 0.8 | < 0.5 | -13 | -14 | -15 | -15.5 | -16 | -16.5 | -17 | -17.5 | -18 | -18 | -18 | -18 |
| < 0.5 | < 0.5 | -13 | -14 | -15 | -15.5 | -16 | -16.5 | -17 | -17.5 | -18 | -18 | -18 | -18 |

### Summary of new mutation risk between 3-6 months, and after 6 months if viral load at t-1 > 4 log copies/ml

This table applies to patients for whom it has been between 3 and 6 months since starting their current period of continuous therapy, as well as for patients whom it has been more than 6 months since their current period of continuous therapy but who have a high viral load (e.g. due to previous poor adherence). The numbers given in the table below correspond to the ‘new mutation factor’, which is a number that is multiplied by the viral load (mean of values at ($t-1)$ to $t$). The resulting probability, *newmut{t}* is used when assessing whether a new mutation or mutations have arisen (see section 3.4.1).

|  |  | **Number of active drugs** | | | | | | | | | | | |
| --- | --- | --- | --- | --- | --- | --- | --- | --- | --- | --- | --- | --- | --- |
| **‘Effective adherence’ between *t-2* & *t-1*** | **‘Effective adherence’ between *t-1* & *t*** | **3** | **2.75** | **2.5** | **2.25** | **2** | **1.75** | **1.5** | **1.25** | **1** | **0.75** | **0.5** | **0.25** |
| > 0.8 | > 0.8 | 0.002 | 0.01 | 0.03 | 0.05 | 0.05 | 0.1 | 0.2 | 0.3 | 0.4 | 0.45 | 0.5 | 0.5 |
| > 0.5, < 0.8 | > 0.8 | 0.002 | 0.01 | 0.03 | 0.05 | 0.05 | 0.1 | 0.2 | 0.3 | 0.4 | 0.45 | 0.5 | 0.5 |
| < 0.5 | > 0.8 | 0.05 | 0.05 | 0.03 | 0.05 | 0.05 | 0.1 | 0.2 | 0.3 | 0.4 | 0.45 | 0.5 | 0.25 |
|  |  |  |  |  |  |  |  |  |  |  |  |  |  |
| > 0.8 | > 0.5, < 0.8 | 0.10 | 0.15 | 0.2 | 0.2 | 0.3 | 0.3 | 0.3 | 0.35 | 0.4 | 0.45 | 0.5 | 0.5 |
| > 0.5, < 0.8 | > 0.5, < 0.8 | 0.10 | 0.15 | 0.2 | 0.2 | 0.3 | 0.3 | 0.3 | 0.35 | 0.4 | 0.45 | 0.5 | 0.5 |
| < 0.5 | > 0.5, < 0.8 | 0.10 | 0.15 | 0.2 | 0.2 | 0.3 | 0.3 | 0.3 | 0.35 | 0.4 | 0.45 | 0.5 | 0.25 |
|  |  |  |  |  |  |  |  |  |  |  |  |  |  |
| > 0.8 | < 0.5 | 0.05 | 0.05 | 0.05 | 0.05 | 0.05 | 0.05 | 0.05 | 0.05 | 0.05 | 0.05 | 0.05 | 0.05 |
| > 0.5, < 0.8 | < 0.5 | 0.05 | 0.05 | 0.05 | 0.05 | 0.05 | 0.05 | 0.05 | 0.05 | 0.05 | 0.05 | 0.05 | 0.05 |
| < 0.5 | < 0.5 | 0.05 | 0.05 | 0.05 | 0.05 | 0.05 | 0.05 | 0.05 | 0.05 | 0.05 | 0.05 | 0.05 | 0.05 |

### Summary of viral load (mean change from viral load max), CD4 count change (mean change between t-1 and t), after 6 months, where viral load at t-1 < 4 log copies/ml.

Summary of viral load (mean change from viral load max), CD4 count change (mean change between t-1 and t), and new mutation risk after 6 months, where viral load at t-1 < 4 logs. For viral load this is the mean of a Normal distribution with standard deviation 0.2, from which the patient's value/change is sampled. For the CD4 count patients vary in their underlying propensity for CD4 rise on ART (given by given by *pt_cd4_rise_art*) and the CD4 count change given here is multiplied by this factor. For the new mutation number, this is a number that is multiplied by the viral load (mean of values at ($t-1)$ to $t$). The resulting probability, *newmut{t}* is used when assessing whether a new mutation or mutations have arisen (see section 3.4.1).

|  |  | **Number of active drugs** | | | | | | | | | | | |
| --- | --- | --- | --- | --- | --- | --- | --- | --- | --- | --- | --- | --- | --- |
|  | **‘Effective**  **adherence’ between *t-1* & *t*** | **3** | **2.75** | **2.5** | **2.25** | **2** | **1.75** | **1.5** | **1.25** | **1** | **0.75** | **0.5** | **0.25** |
| **Viral load (log change from *vmax*)** | > 0.8 | 0.5 | 0.9 | 1.2 | 1.6 | -2.5 | -2.0 | -1.4 | -1.15 | -0.9 | -0.75 | -0.6 | -0.3 |
|  | > 0.5, < 0.8 | 1.2 | 1.2 | 1.2 | 1.4 | -1.2 | -1.0 | -0.7 | -0.6 | -0.5 | -0.4 | -0.3 | -0.1 |
|  | < 0.5 | -0.5 | -0.4 | -0.3 | -0.25 | -0.2 | -0.2 | -0.1 | -0.1 | -0.1 | -0.1 | -0.1 | 0 |
|  |  |  |  |  |  |  |  |  |  |  |  |  |  |
| **CD4 count change (*t-1* to *t*)** | > 0.8 | +30 | +28 | +25 | +23 | +21 | +19 | +3 | -5 | -9 | -10.5 | -12 | -12 |
|  | > 0.5, < 0.8 | +15 | +13 | +10 | +8 | -4.5 | -7.5 | -10 | -12 | -13 | -14 | -15 | -15 |
|  | < 0.5 | -13 | -14 | -15 | -15.5 | -16 | -16.5 | -17 | -17 | -18 | -17 | -17 | -17 |
|  |  |  |  |  |  |  |  |  |  |  |  |  |  |
| **New mutation risk (x log viral load)** | > 0.8 | 0.002 | 0.01 | 0.03 | 0.08 | 0.1 | 0.15 | 0.2 | 0.3 | 0.4 | 0.45 | 0.5 | 0.5 |
|  | > 0.5, < 0.8 | 0.15 | 0.18 | 0.2 | 0.25 | 0.3 | 0.3 | 0.3 | 0.35 | 0.4 | 0.45 | 0.5 | 0.5 |
|  | < 0.5 | 0.05 | 0.05 | 0.05 | 0.05 | 0.05 | 0.05 | 0.05 | 0.05 | 0.05 | 0.05 | 0.05 | 0.05 |

### Changes in viral load, CD4 count and new mutation risk if the number of active drugs in current regimen = 0

For 0 active drugs, these are the changes regardless of time from start of regimen.

|  |  | **Number of active drugs** |
| --- | --- | --- |
|  | **‘Effective**  **adherence’ between *t-1* & *t*** | **0** |
| **Viral load (log change from *vmax*)** | > 0.8 | -0.3 |
|  | > 0.5, < 0.8 | -0.1 |
|  | < 0.5 | 0 |
|  |  |  |
| **CD4 count change (*t-1* to *t*)** | > 0.8 | -15 |
|  | > 0.5, < 0.8 | -17 |
|  | < 0.5 | -18 |
|  |  |  |
| **New mutation risk (x log viral load)** | > 0.8 | 0.5 |
|  | > 0.5, < 0.8 | 0.5 |
|  | < 0.5 | 0.05 |

### Factors which affect the CD4 count rise

There are a number of effects and factors which are taken into account before the final CD4 count rise per 3 months is determined. There is a maximum CD4 count achievable when on ART, which is fixed for each patient, *cmax*. This estimate is based on observed CD4 counts in HIV-negative people[44, 45].

#### Variable patient-specific tendency for CD4 count rise on ART

Patients are assumed to vary in their underlying propensity for CD4 count rise whilst on ART. Each person is given a value for their propensity, ‘*pt_CD4_rise_art*’. This value is fixed and remains constant for the individual over time and is the factor by which the CD4 count change is multiplied by in sections 3.3.2, 3.3.5 and 3.3.7.

To reflect the fact that the rate of CD4 count increase on ART tends to diminish with time[42, 46], for those with *pt_CD4_rise_art*’ > 1, this factor is modified by a factor 0.67 after 1 year of continuous treatment and by a factor of 0.5 after 3 years of continuous treatment.

#### Accelerated rate of CD4 count loss if PI not present in regimen

The rate of change in CD4 count in people on failing regimens is largely based on data from the PLATO collaboration, for which patients were mainly on regimens containing a PI[47]. If the regimen does not contain a PI, the change in CD4 count per 3 months is modified (additive effect) by *poorer_cd4_rise_on_failing_nnrti*. This applies regardless of viral load level, so PIs are assumed to lead to a more beneficial CD4 count change than NNRTIs.

#### Effect of age and gender

Being female and younger age is associated with larger CD4 count rise while on ART[48-50] (also based on unpublished analyses in COHERE). The CD4 count rise per 3 months is +2 higher if female and $age\left\{ t \right\}\times0.3$ higher per one year younger.

#### Variability in individual (underlying) CD4 counts for people on ART

Once the mean of the underlying CD4 count is obtained as described above for people on ART, to obtain the CD4 count, variability, *sd_cd4*, is added on the square root scale. The estimate was based on unpublished analyses.

### Viral load and CD4 count changes during ART interruption

Viral load returns to previous maximum viral load (*vmax*) in 3 months and adopts natural history changes thereafter.

CD4 rate of decline returns to natural history changes (i.e. those in ART-naïve patients) after 9 months, unless the count remains > 200 cells/mm^3^ above the CD4 count nadir, *cmin{t}*.

Rate of CD4 count decline depends on current viral load:

| **Time off ART** | **Current viral load (log copies/ml)** | **Distribution of change in CD4 count (cells/mm^3^)** |
| --- | --- | --- |
| 3 months, or >3 months and CD4 count in previous period is >300 above the minimum CD4 count to date | VL > 5 | Normal (-200,10) |
|  | 4.5 ≤ VL < 5 | Normal (-160,10) |
|  | VL < 4.5 | Normal (-120,10) |
| 6 months | VL > 5 | Normal (-100,10) |
|  | 4.5 ≤ VL < 5 | Normal (-90,10) |
|  | VL < 4.5 | Normal (-80,10) |
| 9 months | VL > 5 | Normal (-80,10) |
|  | 4.5 ≤ VL < 5 | Normal (-70,10) |
|  | VL < 4.5 | Normal (-60,10) |

If these changes lead to $c\left\{ t \right\}<cmin\{t\}$ then $c\left\{ t \right\}=cmin\left\{ t \right\}$, i.e. current CD4 count is set as the CD4 count nadir.

These values are broadly based on evidence from a number of analyses of the effects of ART interruption[20-22, 51-59].

## Resistance

### Modelling resistance

The choice of mutations to include reflects a balance between the desire to capture important specific effects and the need to limit the complexity of the model and the number of variables simulated. The IAS-USA resistance guidelines provided the basis for choice of mutations[60].

We do not specify the mutated amino acid for each position; it is assumed that for a given codon position, the mutations considered are those that confer resistance (e.g. for M184 this is I or V). The exceptions to this are the mutations at codon 50 of protease inhibitors.

Resistance mutations can be present in majority or minority virus and this is also reflected in the model. Unlike all other resistance mutations, M184 is assumed not to persist in majority virus after HIV infection; although like all other mutations, it does persist as minority virus.

### Accumulation of resistance mutations

*newmut{t}* (see sections 3.3.2, 3.3.3, 3.3.6 and 3.3.7) is a probability used to indicate the level of risk of new mutations arising in a given 3 month period. If this chance comes up in a given 3 month period (determined by sampling from the binomial distribution) then the following criteria operate (presented per drug class):

| **Resistance mutation** | **Probability**  **of arising** | **Conditions** |
| --- | --- | --- |
| M184 | 50% | if (on 3TC) |
| # TAMS increases by 1 | 20% | if (on ZDV or D4T) and (not on 3TC nor FTC) |
|  | 12% | if (on ZDV or D4T) and (on 3TC or FTC) |
| # TAMS increases by 2 | 1% | if (on ZDV or D4T) and (not on 3TC nor FTC) |
|  | 1% | if (on ZDV or D4T) and (on 3TC or FTC) |
| K65 | 2% | if (on TEN or ABA or DDI) and (on ZDV or D4T) |
|  | 10% | If (on TEN or ABA or DDI) and (not on ZDV nor D4T) |
| L74 | 1% | if (on DDI or DDC or ABA) |
| Q151 | 2% | if (on DDI or D4T or ZDV or ABA) |
| Other new NRTI mutations | 10% | if on NNU |
| K103 | 20% | If on NEV |
|  | 60% | If on EFA |
| Y181 | 40% | If on NEV |
|  | 10% | If on EFA |
|  | 30% | If on ETR |
| G190 | 20% | If on NEV |
|  | 10% | If on EFA |
| Etravirine mutation | 10% | If on ETR |
| D30 | 15% | if on NEL |
| V32 | 4% | if on LPR |
| M46 | 12% | If (on IND) and (year of infection < July 2000) |
|  | 4% | If (on IND) and (year of infection ≥ July 2000) |
|  | 12% | If on RIT |
| I47 | 4% | If on LPR |
| G48 | 60% | If (on SAQ) and (year of infection < 1997) |
|  | 12% | If (on SAQ) and (1997 ≤ year of infection < 1999) |
|  | 4% | If (on SAQ) and (year of infection ≥ 1999) |
| I50V | 12% | If (on AMP) and (year of infection < July 2003) |
|  | 4% | If (on AMP) and (year of infection ≥ July 2003) |
|  | 2% | If on DAR |
| I50L | 3% | If on TAZ |
| I54 | 2% | If on DAR |
| L76 | 2% | If on DAR |
| V82 | 12% | If (on IND) and (year of infection < July 2000) |
|  | 4% | If (on IND) and (year of infection ≥ July 2000) |
|  | 12% | If on RIT |
|  | 4% | If on LPR |
| I84 | 12% | If (on IND) and (year of infection < July 2000) |
|  | 4% | If (on IND) and (year of infection ≥ July 2000) |
|  | 12% | If on RIT |
|  | 12% | If (on AMP) and (year of infection < July 2003) |
|  | 4% | If (on AMP) and (year of infection ≥ July 2003) |
|  | 3% | If on TAZ |
|  | 2% | If on DAR |
| N88 | 3% | If on TAZ |
| L90 | 60% | If (on SAQ) and (year of infection < 1997) |
|  | 12% | If (on SAQ) and (1997 ≤ year of infection < 1999) |
|  | 4% | If (on SAQ) and (year of infection ≥ 1999) |
|  | 15% | If on NEL |
| CCR5 inhibitor mutations | 7% | If on MAR |
| Primary integrase inhibitor mutations | 20% | If on RAL |
| Secondary integrase inhibitor mutations | 20% | If on RAL |
| Fusion inhibitor mutations | 20% | If on ENF |

We assume a different probability of resistance mutation accumulation depending on whether the PI would be boosted or not (which for simplicity, we assume it depends entirely on the current calendar year).

These values are chosen, in conjunction with values of *newmut{t}*, to provide estimates of accumulation of specific classes of mutation consistent with those observed in clinical practice[39, 40]. They reflect a greater propensity for some mutations to arise than others. This probably relates to the ability of the virus to replicate without the mutations (e.g. probably very low in the presence of 3TC for virus without M184V) as well as the replicative capacity of virus with the mutations. Over time as more data accumulate it may be possible improve these estimates of rates of accumulation of specific mutations.

### Loss of acquired mutations from majority virus

It is assumed that mutations tend to be lost from majority virus with a certain probability from 3 months after stopping to take a drug that selects for that mutation. The probability of losing mutations per 3 months (from 3 months after stopping) is summarised in the table below. These values were chosen based on evidence from studies in people interrupting ART[61-66]. Note that these probabilities all relate to people who have started ART and are not about persistence of transmitted mutations (which is currently assumed to be indefinite, except for M184V).

| **Resistance mutation** | **Probability of loss (per 3 months)** |
| --- | --- |
| M184V | 80% |
| L74V | 60% |
| K65R | 60% |
| Q151M | 60% |
| TAMS (lose all) | 40% |
| Other new NRTI mutations | 40% |
| K103N | 20% |
| Y181C | 20% |
| G190A | 20% |
| Etravirine mutation | 20% |
| D30 | 20% |
| V32 | 20% |
| M46 | 20% |
| I47 | 20% |
| G48 | 20% |
| I50V | 20% |
| I50L | 20% |
| I54 | 20% |
| L76 | 20% |
| V82 | 20% |
| I84 | 20% |
| N88 | 20% |
| L90 | 20% |
| CCR5 inhibitor mutations | 20% |
| Primary integrase inhibitor mutations | 20% |
| Secondary integrase inhibitor mutations | 20% |
| Fusion inhibitor mutations | 60% |

### “Regaining” mutations in majority virus after restarting ART

Mutations previously present are regained when one of the corresponding drugs listed above is restarted.

### Determination of level of resistance to each drug

| **Resistance mutation** | **Drug** | **Level of resistance**  **(1=full resistance)** | **Condition** |
| --- | --- | --- | --- |
| M184 | 3TC or FTC | 0.75 |  |
|  | ABA | 0.25 |  |
| 1-2 TAMS | ZDV or D4T | 0.5 | No 3TC or FTC in regimen |
|  | ZDV or D4T | 0.25 | 3TC or FTC in regimen and ever had M184V |
|  | ZDV or D4T | 0.5 | 3TC or FTC in regimen and never had M184V |
|  | ABA | 0.25 |  |
| 2-3 TAMS | TEN | 0.5 | No 3TC or FTC in regimen |
|  | TEN | 0.25 | 3TC or FTC in regimen and ever had M184V |
|  | TEN | 0.5 | 3TC or FTC in regimen and never had M184V |
| 3-4 TAMS | ZDV or D4T | 0.75 | No 3TC or FTC in regimen |
|  | ZDV or D4T | 0.5 | 3TC or FTC in regimen and ever had M184V |
|  | ZDV or D4T | 0.75 | 3TC or FTC in regimen and never had M184V |
|  | ABA | 0.5 |  |
| 3 or more TAMS | DDI | 0.5 |  |
| 4 or more TAMS | TEN | 0.75 | No 3TC or FTC in regimen |
|  | TEN | 0.5 | 3TC or FTC in regimen and ever had M184V |
|  | TEN | 0.75 | 3TC or FTC in regimen and never had M184V |
| 5 or more TAMS | ZDV or D4T | 1.0 | No 3TC or FTC in regimen |
|  | ZDV or D4T | 0.75 | 3TC or FTC in regimen and ever had M184V |
|  | ZDV or D4T | 0.75 | 3TC or FTC in regimen and never had M184V |
|  | ABA | 0.75 |  |
| Q151 | ZDV or D4T or ABA or DDI | 0.75 |  |
|  | ABA | 0.75 |  |
| K65 | D4T | 0.5 |  |
|  | TEN or ABA or DDI | 0.75 |  |
| L74 | ABA | 0.5 |  |
|  | DDI | 0.75 |  |
| Other new NRTI mutations | NNU | 0.75 |  |
| K103 | NEV or EFA | 1.0 |  |
| Y181 | NEV | 1.0 |  |
|  | EFA | 0.75 |  |
|  | ETR | 0.5 |  |
| G190 | NEV | 1.0 |  |
|  | EFA | 0.75 |  |
|  | ETR | 0.25 |  |
| Etravirine mutations | ETR | 0.5 |  |
|  | ETR | 1.0 | ever had Y181 or G190 |
| D30 | NEL | 1.0 |  |
| I47 | LPR | 0.75 |  |
| G48 | SAQ | 0.75 |  |
| I50V | AMP | 0.75 |  |
| I50L | TAZ | 1.0 |  |
| V82 | AMP | 0.25 | Never had I50V |
|  | RIT | 1.0 |  |
| I84 | AMP | 0.75 |  |
|  | AMP | 0.25 | Never had I50V |
|  | RIT | 1.0 |  |
|  | TAZ | 1.0 |  |
| N88 | TAZ | 1.0 |  |
| L90 | SAQ | 0.5 |  |
|  | NEL | 1.0 |  |
| 1 or 2 of (M46, V82, I84) | IND | 0.5 |  |
| All of (M46, V82, I84) | IND | 0.75 |  |
| 1 of (G48, I84) | TAZ | 0.5 |  |
| 1 of (G48, I84) | TAZ | 1.0 | Ever had at least 2 of (V32, M46, I54, V82, L90) |
| Both of (G48, I84) | TAZ | 1.0 |  |
| 1 or 2 or 3 of (V32, M46, I54, V82, L90) | TAZ | 0.5 |  |
| At least 4 of (V32, M46, I54, V82, L90) | TAZ | 1.0 |  |
| 1 of (V32, L76, V82) | LPR | 0.25 | Never had I47 |
| 2 of (V32, L76, V82) | LPR | 0.5 | Never had I47 |
| 3 of (V32, L76, V82) | LPR | 0.75 | Never had I47 |
| All of (V32, I47, L76, V82) | LPR | 1.0 |  |
| 2 of (V32, I47, I50V, I54, L76, I84) | DAR | 0.25 |  |
| 3 of (V32, I47, I50V, I54, L76, I84) | DAR | 0.5 |  |
| At least 4 of (V32, I47, I50V, I54, L76, I84) | DAR | 0.75 |  |
| 4 of (M46, V82, I84, L90) | SAQ or RIT or IND or NEL or AMP or LPR | Max(level of resistance as above in this table, 0.5) |  |
| 2 or 3 of (M46, V82, I84, L90) | SAQ or RIT or IND or NEL or AMP or LPR | Max(level of resistance as above in this table, 0.25) |  |
| CCR5 inhibitor mutations | MAR | 1.0 |  |
| Primary integrase inhibitor mutations | RAL | 0.75 |  |
|  | RAL | 1.0 | Ever had secondary integrase inhibitor mutation |
| Secondary integrase inhibitor mutations | RAL | 0.25 |  |
| Fusion inhibitor mutations | ENF | 1.0 |  |

These rules approximately follow the interpretation systems for conversion of mutations present on genotypic resistance test into a predicted level of drug activity (or, equivalently, of resistance). Currently interpretation systems differ in their prediction of activity for some drugs.

### Calculation of activity level of drug

Every drug is treated as being equally potent because virologic efficacy depends only on number of active drugs, not which specific drugs they are that are active. In reality, drugs differ in potency but to our knowledge no reliable estimates are available to use. The exception is for boosted-PI drugs which are assumed to have double potency of all other drugs.

The number of active drugs in the regimen at time t, *nactive{t},* is given by 1 – level of resistance, as described in section 3.4.5. Activity levels of each drug in the regimen are summed to give the total number of active drugs.

## Toxicity

Toxicities including gastrointestinal symptoms, rash, acute hepatoxicity, CNS toxicity, lipodystrophy, hypersensitivity reaction, peripheral neuropathy and nephrolithiasis can occur with certain probability when the individual is on certain specific drugs. These probabilities are based broadly on evidence from trials and cohort studies, although there are no common definitions for some conditions which complicates this. All toxicity variables are binary, i.e. if the individual develops a certain toxicity in a given 3-month period, it takes the value 1, otherwise 0.

### Incidence of new current toxicity

All individuals do not have any toxicity at the start of simulation (i.e. point of infection). Summarised below is the percentage probability of developing a new current toxicity in any given 3-month period. Other toxicity covers the stopping of new drugs with unknown adverse event profiles.

| **Toxicity** | **Drug** | **Risk of development per 3 months** | **Probability of continuation if pre-existing** |
| --- | --- | --- | --- |
| Nausea | TAZ, DAR | 1% (5-fold higher in 1^st^ year) | 50% |
|  | ZDV, IND, SAQ, DDI, AMP, LPR | 3% (5-fold higher in 1^st^ year) | 50% |
|  | RIT | 50% | 50% |
| Diarrhoea | NEL | 7% (2.5-fold higher in 1^st^ year) | 50% |
|  | AMP, DDI, SAQ, RIT | 5% (2.5-fold higher in 1^st^ year) | 50% |
|  | LPR | 2% (2.5-fold higher in 1^st^ year) | 50% |
|  | TAZ, DAR | 1% (2.5-fold higher in 1^st^ year) | 50% |
| Rash | EFA | 3% (if not on EFA 6 months ago) |  |
|  | NEV | 10% (if not on EFA 6 months ago) |  |
| CNS toxicity | EFA | 10% (Been on current regimen <1 year) | 80% if been on current regimen <1 year. 90% if been on current regimen ≥1 year |
| Lipodystrophy | D4T | 5% | 100% |
|  | ZDV | 1.5% | 100% |
| Hypersensitivity reaction | ABA | 10% (Been on current regimen <3 months) |  |
| Peripheral neuropathy | D4T, DDC | 2% (1.5-fold higher in 1^st^ year) | 100% (if remain on d4T or ddC) |
|  | DDI | 1% (1.5-fold higher in 1^st^ year) | 100% (if remain on ddI) |
| Acute hepatitis | NEV | 2% (one off risk in 1^st^ and 2^nd^ 3 month periods) |  |
| Nephrolithiasis | IND | 25% (1.5-fold higher in 1^st^ year) |  |
| Anaemia | ZDV | 3% (1.5-fold higher in 1^st^ year) | 20% |
| Headache | ZDV | 10% (1.5-fold higher in 1^st^ year) | 40% |
| Pancreatitis | D4T, DDI | 0.5% (1.5-fold higher in 1^st^ year) | 100% |
| Lactic acidosis | D4T, DDI | 1% |  |
|  | ZDV, ABA, TEN | 0.01% |  |
| Renal dysfunction | TEN | 0.35% |  |
| Other toxicity | Any drug | 3% (1.5-fold higher in 1^st^ year) |  |

### Switching of drugs due to toxicity

If toxicity is present then individual drugs may be switched due to toxicity. In most cases, the switch is to another in the same class, if such a drug (that has not been previously failed nor stopped due to toxicity) is available. This will vary by setting and availability of alternative drugs.

## Risk of clinical disease and death

The choices of parameter estimates in this section are broadly based on references[47, 67-70]. Factors were chosen to provide results consistent with observed data, including the incubation period for death and the time from AIDS to death in untreated people[12, 13, 71-73].

### Parameter values and distributions

| **Parameter** | | **Value (or distribution) where applicable** |
| --- | --- | --- |
| **Variable name in program** | **Description** |  |
| *Base_rate* | Rate of AIDS (differs by CD4 count) |  |
| *Pcp_use_prob* | Probability of PCP use per 3 months if CD4 <200 cells/mm^3^ | 0.9 |
| *fold_incr_cdcb* | Fold increase in risk of CDC B symptoms compared to AIDS | 5 |
| *Fold_decr_hivdeath* | Fold decrease in risk of HIV-related death, compared to AIDS | 0.25 |

### Occurrence of AIDS

The rate of AIDS, defined by the variable, *base_rate*, according to (most recent) CD4 count is as follows:

| **CD4 count** | **Rate (per year)** |  | **CD4 count** | **Rate (per year)** |
| --- | --- | --- | --- | --- |
| > 650 | 0.002 |  | 150 - 174 | 0.10 |
| 500 - 649 | 0.010 |  | 125 - 149 | 0.13 |
| 450 - 499 | 0.013 |  | 100 - 124 | 0.17 |
| 400 - 449 | 0.016 |  | 90 - 99 | 0.20 |
| 375 - 399 | 0.020 |  | 80 - 89 | 0.23 |
| 350 - 374 | 0.022 |  | 70 - 79 | 0.28 |
| 325 - 349 | 0.025 |  | 60 - 69 | 0.32 |
| 300 - 324 | 0.030 |  | 50 - 59 | 0.40 |
| 275 - 299 | 0.037 |  | 40 - 49 | 0.50 |
| 250 - 274 | 0.045 |  | 30 - 39 | 0.80 |
| 225 - 249 | 0.055 |  | 20 -29 | 1.10 |
| 200 - 224 | 0.065 |  | 10-19 | 1.80 |
| 175 - 199 | 0.080 |  | < 10 | 2.50 |

There is an independent effect of viral load, age, being on PCP prophylaxis and being on ART.

**Independent effect of viral load**

| **Viral load (log)** | **Multiply rate by** |
| --- | --- |
| < 3 | 0.2 |
| 3 - 3.99 | 0.3 |
| 4 - 4.49 | 0.6 |
| 4.5 - 4.99 | 0.9 |
| 5 - 5.49 | 1.2 |
| >= 5.5 | 1.6 |

**Independent effect of age**

Rates increase with age. Multiply rate by a further factor of (age/38)^1.2^.

**Independent effect of PCP prophylaxis**

If patient is on PCP prophylaxis, multiply rate by a further factor of 0.8.

There is a 90% chance, given by *pcp_use_prob*, that the patient will be on PCP prophylaxis in a given 3-month period if they have a measured CD4 count <200 cells/mm^3^.

**Independent effect of being on ART**

The rate is multiplied by a further factor of 0.9, 0.85 and 0.8 if on a single drug, 2 drug or 3 drug regimen respectively. These factors reflect that being on HAART has a positive effect on risk of AIDS and death, independent of latest CD4 count and viral load.

### Occurrence of WHO 3 diseases

The rate of occurrence of CDC category B symptoms is as for AIDS, but *fold_incr_cdcb* higher.

In a given 3-month period, if a patient is diagnosed with an ADC, they are also diagnosed with AIDS (if they haven’t been diagnosed with AIDS previously). If a patient has an ADC, they have a 5% chance of lymphoma or they have a 2% chance of progressive multifocal leukoencephalopathy (PML), if their CD4 count is less than 50. We have singled out lymphoma and PML because these severe AIDS defining conditions substantially increase the rate of death[74].

### Occurrence of HIV-related deaths

The rate of occurrence of HIV-related deaths is as for AIDS, but *fold_decr_hivdeath* higher.

The occurrence of deaths, which are explicitly not due to non-HIV causes, is closely related to CD4 count. Some of these deaths however, although related to CD4 count, will not be HIV-related (e.g. other cancers). Therefore of the CD4-related deaths, a proportion (15%) will be classified as non-HIV deaths, and the remaining 85% will be classified as HIV-related deaths.

**Independent effect of lymphoma/PML**

If lymphoma has occurred anytime in the last 6 months, multiply rate by 5. If PML has occurred anytime in the last 6 months, the rate per year is 0.53.

### Occurrence of non-HIV-related deaths

Rates from country-specific national mortality statistics (gender-specific) for 2011 are used.

There is increasing evidence that people with HIV infection itself may have a raised risk of common clinical conditions such as non-AIDS cancers, renal and liver disease and cardiovascular diseases[75-80]. Data from observational studies suggest that there is a modest increased risk of death for HIV-positive people with CD4 count greater than 500/mm^3^, compared to the general population, of the order of approximately 1.5[81, 82]. Hence, we also assumed that there was a 1.5-fold increased rate of all non-HIV causes of death throughout life.

**Effect of smoking**

Smokers experience 1.5-fold increased rate of non-HIV deaths. Non-smokers experience 0.75-fold increased rate of non-HIV deaths (i.e. decreased risk of death). This is consistent with a two-fold increase in all-cause mortality associated with smoking[83].

# Model fits

## Incubation period to AIDS and death from seroconversion (no ART)

### Incubation period to AIDS (no ART) stratified by sex and race (black vs white). Observed data from reference [13].

### Incubation period to AIDS (no ART) stratified by age. Dotted line shows modelled data. Observed data from reference [13].


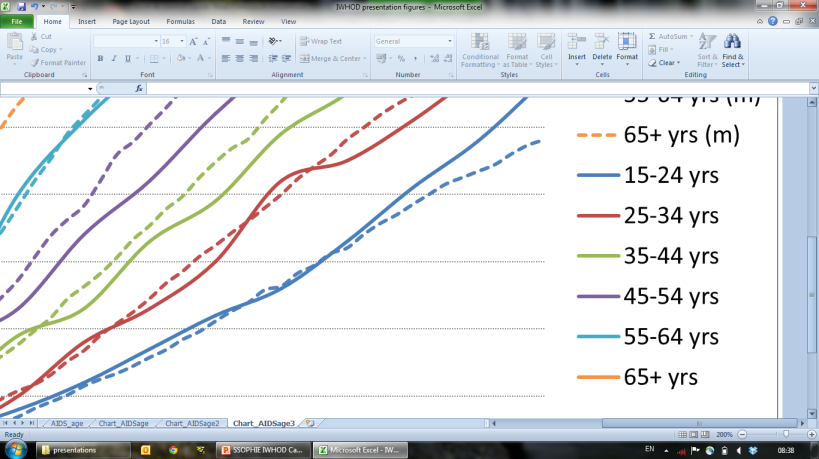


### Incubation period to death (no ART) stratified by sex and race (black vs white). Observed data from reference [13].

### Incubation period from AIDS to death (no ART). Observed data from reference [73].

### Time to CD4 count <200, <350, <500 cells/mm^3^ (no ART). Observed data from reference [84].


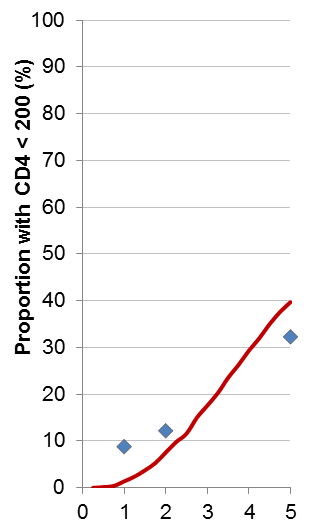

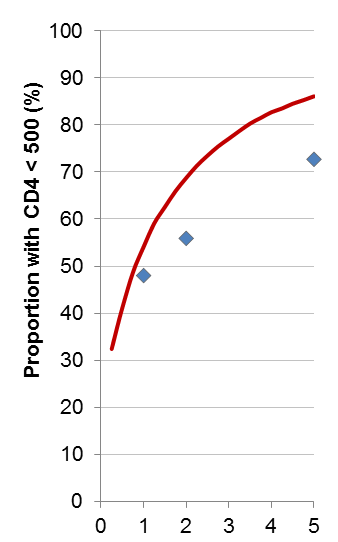


## Other model fits relating to the natural history of HIV

### Viral load set point and initial CD4 count (after primary infection). Observed data from reference [85]

|  | Observed | Model |
| --- | --- | --- |
| Median VL set point | 4.5 | 4.0 (IQR: 3.6-4.3) |
| Median initial CD4 count | 570 | 565 (IQR: 485-641) |

### Association between viral load measured close to seroconversion (between 6-24 months) and risk of AIDS, adjusting for CD4 count and age. Observed data from reference [8].

|  | Adjusted Relative Hazard (95% CI) | |
| --- | --- | --- |
|  | **Observed** | **Model** |
| Viral load (per 0.5 log higher) | 1.87 (1.58 – 2.20) | 2.13 (2.07 – 2.18) |
| CD4 count (per 100 cells/mm^3^ lower) | 1.12 (1.02 – 1.24) | 1.16 (1.14 – 1.18) |
| Age (per 10 years older) | 1.19 (0.96 – 1.47) | 1.49 (1.46 – 1.52) |

### Cumulative 6-year risk of AIDS by CD4 count and viral load and age in the absence of ART. Observed data from reference [12].

| CD4 count | Viral load | Observed | Model |
| --- | --- | --- | --- |
| < 350 | < 1500 - (low n) | - | - |
|  | 1501- 7000 | 19% | 47% |
|  | 7001- 20000 | 42% | 65% |
|  | 20001- 55000 | 73% | 83% |
|  | > 55000 | 92% | 93% |
| 350-500 | < 1500 - (low n) | - | - |
|  | 1501- 7000 | 22% | 18% |
|  | 7001- 20000 | 40% | 33% |
|  | 20001- 55000 | 57% | 57% |
|  | > 55000 | 78% | 75% |
| > 500 | < 1500 - (low n) | 5% | 0% |
|  | 1501- 7000 | 15% | 6% |
|  | 7001- 20000 | 26% | 17% |
|  | 20001- 55000 | 48% | 32% |
|  | > 55000 | 67% | 69% |

* Viral load values used in MACS may need to be multiplied by

~ 2 to approximate to more commonly used Roche assay levels.

### Median CD4 count at diagnosis of AIDS and at death (No ART). Observed data from reference [71]

|  | Median (IQR) CD4 count, cells/mm^3^ | |
| --- | --- | --- |
|  | **Observed** | **Model** |
| At AIDS | ~40 | 49 (17-120) |
| At death | ~0 | 9 (2-36) |

## Model fits relating to the effect of ART

### 3 year percent risk of AIDS after start of ART by baseline CD4 / viral load (age < 50, non-IDU, AIDS-free). Observed data from reference [86].

| Baseline viral load | Baseline CD4 count | Observed | Model |
| --- | --- | --- | --- |
| < 100,000 | < 50 | 16% | 15% |
|  | 50-99 | 12% | 11% |
|  | 100-199 | 9% | 10% |
|  | 200-349 | 5% | 5% |
|  | > 350 | 3% | 3% |
| > 100,000 | < 50 | 20% | 20% |
|  | 50-99 | 16% | 10% |
|  | 100-199 | 12% | 13% |
|  | 200-349 | 6% | 9% |
|  | > 350 | 4% | 0% |

### % with virologic failure (viral load > 500 copies/mL / on ART) by time from start of HAART (patients starting with PI/r or NNRTI regimen). Observed data from reference [87].

Observed data may be overestimates due to some unrecognised stopping of ART

### Effect of HAART vs. no therapy on risk of AIDS and death. Observed data from reference [88].

Simulated trial with 5 years follow up.

|  | Observed | Model |
| --- | --- | --- |
| Relative hazard of AIDS (HAART vs. no therapy) | 0.1 | 0.17 |

### Rate of viral rebound in people on 1st line HAART and with viral load < 50 copies/mL. Observed data from reference [89].

|  | Observed | Model |
| --- | --- | --- |
| Rate per 100 person -years | 3-6 | 4.8 |

### Median CD4 count change at 3 years from start of HAART. Observed data from reference [38].

|  | Observed | Model |
| --- | --- | --- |
| Median CD4 count change | 273 | 228 |

Note that in the above example, the observed data is based on results from one clinical trial. Data on CD4 count increases from starting HAART seem to vary hugely.

### Discontinuation of drugs in initial HAART regimen. Observed data from reference [90].

Time from start of ART to discontinuation (for any reason) of at least one drug in initial regimen.

| Years from start of HAART | Observed | Model  (estimates for 1996-2001 inclusive) |
| --- | --- | --- |
| 1 | 30% | 29% |
| 2 | 45% | 42% |
| 3 | 62% | 52% |
| 4 | 73% | 58% |

### Percent with triple class virologic failure by years from start of HAART (patients naïve before HAART). Observed data from reference [91].

| Years from start of HAART | Observed | Model  (estimates based on ART start years 1997-2003 inclusive) |
| --- | --- | --- |
| 1 | 1% | 0% |
| 2 | 3% | 2% |
| 3 | 4% | 4% |
| 4 | 7% | 6% |
| 5 | 9% | 8% |
| 6 | 12% | 9% |

### Triple class failure (those with triple class failure before 2001). Observed data from reference [47].

|  | Observed | Model |
| --- | --- | --- |
| % ever previously with viral load < 500 | 50% | 76% |

At time of triple class failure:

|  | Observed | Model |
| --- | --- | --- |
| Median (IQR) viral load | 4.5 (3.9 – 5.0) | 3.8 (3.4 – 4.3) |
| Median (IQR) CD4 count | 199 (97 – 340) | 108 (38 – 212) |
| Median (IQR) CD4 count nadir | 65 (17 – 169) | 20 (0 – 86) |
| Duration of ART (years) | 4.7 (3.2 – 6.7) | 5.3 (4.0 – 8.0) |
| % starting ART with >3 drugs | 15% | 23% |

### Percent with triple class virological failure by years from start of HAART (patients naïve before HAART). Observed data from reference [92].

| Years from start of HAART | Observed | Model  (estimates based on ART start years 1998-2008 inclusive) |
| --- | --- | --- |
| 5 | 3.4% | 6.9% |
| 9 | 8.6% | 13.1% |

### Risk of death after triple class virologic failure. Observed data from reference [47].

| Years from triple class failure  (triple class failure occurring before 2002) | Observed | Model |
| --- | --- | --- |
| 1 | 5% | 7% |
| 2 | 10% | 12% |
| 3 | 15% | 15% |
| 4 | 21% | 16% |

## Model fits relating to resistance

### Risk of resistance mutations (and virologic failure) after start of ART (patients starting with PI/r or NNRTI regimen). Observed data from reference [87].

% with at least one resistance mutation (and virologic failure)

| Years from start of HAART | Observed | Model |
| --- | --- | --- |
| 1 | 4% | 17% |
| 2 | 7% | 24% |
| 3 | 10% | 29% |
| 4 | 12% | 35% |
| 5 | 14% | 40% |
| 6 | 16% | 43% |
| 7 | 19% | 47% |

Observed data underestimates because resistance tests not always performed at virologic failure.

### % with at least one resistance mutation for all three main classes (and virologic failure). Observed data from reference [39].

| Years from start of HAART | Observed | Model |
| --- | --- | --- |
| 2 | 1.0% | 0.4 |
| 4 | 2.7% | 2.2 |
| 6 | 4.1% | 3.8 |

### Risk of resistance mutations after start of ART. Observed data from reference [39].

% with at least one resistance mutation

| Years from start of HAART | 2 | | 4 | | 6 | |
| --- | --- | --- | --- | --- | --- | --- |
|  | Obs. | Model | Obs. | Model | Obs. | Model |
| M184V mutation  (in those starting with 3TC) | 6% | 16% | 13% | 24% | 18% | 30% |
| TAMS  (in those starting with ZDV or d4T) | 4% | 8% | 9% | 14% | 13% | 18% |
| PI mutation  (in those starting with boosted PI regimen) | 3% | 7% | 7% | 9% | - | 10% |
| NNRTI mutation  (in those starting with NNRTI regimen) | 8% | 18% | 14% | 35% | 21% | 43% |

Observed data are likely to be under-estimates as resistance testing is not always performed at virologic failure

### Risk of death after triple class resistance. Observed data from reference [93].

% dead by 3 years (for people with TCR up to 2004.5)

|  | Observed | Model |
| --- | --- | --- |
| % dead by 3 years (for people with TCR up to 2004.5) | 12% | 18% |

# Sensitivity analyses

The effects of varying key assumptions on life expectancy and lifetime costs were explored in multivariable sensitivity analyses. In this analysis, the values of multiple parameters were changed simultaneously.

In the multivariable sensitivity analysis, a total of 10,000 runs of the model were made, each time sampling at random, values for a number of different key parameters in order to generate the distribution of life expectancy. The parameters which were varied, along with the probability distributions which were given in the sensitivity analysis, are shown in Table A. By repeatedly sampling all the variables simultaneously, although in some of the simulations the effect of variables may indeed be cancelling each other out to some extent, in other simulations, it should be capturing many of the relevant parameters at the high and low end of the distributions.

The probability distributions and thus the uncertainty bounds for each parameter were chosen such that even at the boundary values, the parameter was thought to be just plausible. The parameters in Table A were chosen on the basis that there is some uncertainty regarding the assumed value, i.e. some have only limited evidence supporting the choice of value for the parameter and some are purely best guess estimates as, to our knowledge, there is no good quality supporting data.

Probability distributions were generally selected depending on the nature of the variable concerned. Parameters which correspond to probabilities were mainly given Beta distributions, such that the outcomes were restricted to between 0 and 1 inclusive. Parameters which correspond to ratios were mainly given log-normal distributions, such that they are additive on the log scale (and thus multiplicative on the normal scale).

Further to the parameters in Table A, we also varied the adherence pattern for each of the 10,000 runs such that in 60% of the runs, individuals had an underlying tendency to adhere as found in the Model details section above (adherence pattern=2, which is what we estimated from observed data). In the remaining runs, 10% were simulated to have adherence pattern=1, another 10% with adherence pattern=3, another 10% with adherence pattern=4, and the final 10% with adherence pattern=5.

The median life expectancy from this multivariable sensitivity analysis was 69.8 years and the 95% uncertainty bound was (61.5,75.0) years, i.e. Of the 10,000 runs, the estimated life expectancy was between 61.5 and 75.0 years in 95% of the runs.

**Table A:** Parameters distributions used in sensitivity analyses

| **Parameter** | | **Value in**  **base-case analysis** | **Distribution in**  **sensitivity analysis** |
| --- | --- | --- | --- |
| **Variable name in program** | **Description** |  |  |
| *Mean_sqrtcd4_inf* | Initial CD4 count at infection, square root scale | 31 | $Normal(31,2^{2})$ |
| Mean of $V_{set}$ | Mean value of $V_{set}$ | 4.0 | $Normal(4,{0.2}^{2})$ |
| Variance of $V_{set}$ | Variance of variable $V_{set}$ | 0.5 | $Normal(0.5,{0.1}^{2})$ |
| *vmax* | Maximum viral load to that point, log_10_ scale | 6.5 | $Normal(6.5,{0.2}^{2})$ |
| *fx* | Factor adjusting basic rate of natural CD4 count decline | 1.0 | $Log Normal\left( ln1.0,{0.2}^{2} \right)$ |
| *gx* | Factor adjusting basic rate of natural viral load change | 1.0 | $Log Normal\left( ln1.0,{0.2}^{2} \right)$ |
| *Prob_art* | Probability of initiating ART when eligible per 3 months | 0.8 | $Beta(41,11)$ |
| *will_take_enf* | Willingness to take enfuvirtide | 0.85 | $Beta(18,4)$ |
| *rate_inter* | Probability of interruption per 3 months | 0.01 | $Log Normal\left( ln0.01,{0.2}^{2} \right)$ |
| *clinic_not_aware_frac* | Proportion of interruptions where clinic/clinician is not aware of the interruption | 0.3 | $Beta\left( 4,8 \right)$ |
| *rate_restart* | Probability of restarting following interruption per 3 months | 0.6 | $Log Normal\left( ln0.6,{0.2}^{2} \right)$ |
| Pattern of *adhav* and *adhvar* | Distribution of adherence levels for each adherence pattern | 2 | 1: 10%, 2: 60%, 3: 10%, 4: 10%, 5: 10% |
| *pt_cd4_rise_art* | Propensity for CD4 count rise whilst on ART, fixed for each person | $e^{0.2}$ | $Log Normal\left( 0.2,{0.1}^{2} \right)$ |
| *cmax* | Maximum CD4 count to which can return on ART | $e^{6.6}$ | $Log Normal(6.6,{0.25}^{2})$ |
| *poorer_cd4_rise_on_failing_nnrti* | Extent to which CD4 count change is more favourable on a virologically failing bPI-regimen compared with an NNRTI-regimen | 6 | $-6+Normal(3,{0.1}^{2})$ |
| *Nnrti_pi_sa* | Probability of choosing NNRTI or PI-based regimen after any line of failure | 0.5 | $Normal(ln0.5,{0.1}^{2})$ |
| *Mult_newmut* | Multiplicative factor for to modify the probability of acquiring new resistance mutations, *newmut{t}* | 1.0 | $Log Normal(ln1.0,{0.2}^{2})$ |
| *Vf_threshold* | The threshold to decide if someone has virologically failed a regimen, copies/ml | 500 | $Normal(500,{50}^{2})$ |
| *Pcp_use_prob* | Probability of PCP use per 3 months | 0.9 | $Beta\left( 25,5 \right)$ |
| *fold_incr_cdcb* | Fold increase in risk of CDC B symptoms compared to AIDS | 5 | $Log Normal(ln5,{0.2}^{2})$ |
| *Fold_decr_hivdeath* | Fold decrease in risk of HIV-related death, compared to AIDS | 0.25 | $Log Normal(ln5,{0.3}^{2})$ |
| *Fold_change_base_rate* | Fold change in CD4 count-specific rate of occurrence of AIDS, *base_rate* | 1.0 | $Log Normal(ln1.0,{0.2}^{2})$ |
| *Fold_change_ac_death_rate* | Fold change in occurrence of non-HIV causes of death | 1.5 | $Log Normal(ln1.5,{0.2}^{2})$ |

Reference List

1. Phillips AN, Sabin C, Pillay D, Lundgren JD. HIV in the UK 1980-2006: Reconstruction using a model of HIV infection and the effect of antiretroviral therapy. *HIV Med* 2007; **8(8)**:536-546.

2. Bansi L, Sabin C, Delpech V, Hill T, Fisher M, Walsh J*, et al.* Trends over calendar time in antiretroviral treatment success and failure in HIV clinic populations. *HIV Med* 2010; **11(7)**:432-438.

3. Nakagawa F, Lodwick RK, Smith CJ, Smith R, Cambiano V, Lundgren JD*, et al.* Projected life expectancy of people with HIV according to timing of diagnosis. *AIDS* 2012; **26(3)**:335-343.

4. Health Protection Agency. HIV in the United Kingdom: 2012 Report. London: Health Protection Services, Colindale. November 2012. In: 2012.

5. Lyles RH, Munoz A, Yamashita TE, Bazmi H, Detels R, Rinaldo CR*, et al.* Natural history of human immunodeficiency virus type 1 viremia after seroconversion and proximal to AIDS in a large cohort of homosexual men. *J Infect Dis* 2000; **181(3)**:872-880.

6. Pantazis N, Touloumi G. Bivariate modelling of longitudinal measurements of two human immunodeficiency type 1 disease progression markers in the presence of informative drop-outs. *J R Statist Soc C* 2005; **54**:405-423.

7. Sabin CA, Devereux H, Phillips AN, Hill A, Janossy G, Lee CA*, et al.* Course of viral load throughout HIV-1 infection. *J Acquir Immune Defic Syndr* 2000; **23(2)**:172-177.

8. Hubert JB, Burgard M, Dussaix E, Tamalet C, Deveau C, Le Chenadec J*, et al.* Natural history of serum HIV-1 RNA levels in 330 patients with a known date of infection. *AIDS* 2000; **14(2)**:123-131.

9. O'Brien TR, Rosenberg PS, Yellin F, Goedert JJ. Longitudinal HIV-1 RNA levels in a cohort of homosexual men. *J Acquir Immune Defic Syndr* 1998; **18(2)**:155-161.

10. Henrard DR, Phillips JF, Muenz LR, Blattner WA, Wiesner D, Eyster ME*, et al.* Natural history of HIV-1 cell-free viremia. *JAMA* 1995; **274(7)**:554-558.

11. Touloumi G, Pantazis N, Babiker AG, Walker SA, Katsarou O, Karafoulidou A*, et al.* Differences in HIV RNA levels before the initiation of antiretroviral therapy among 1864 individuals with known HIV-1 seroconversion dates. *AIDS* 2004; **18(12)**:1697-1705.

12. Mellors JW, Munoz A, Giorgi JV, Margolick JB, Tassoni CJ, Gupta P*, et al.* Plasma viral load and CD4+ lymphocytes as prognostic markers of HIV-1 infection. *Ann Intern Med* 1997; **126(12)**:946-954.

13. Collaborative Group on AIDS Incubation and HIV Survival and including the CASCADE EU Concerted Action. Time from HIV-1 seroconversion to AIDS and death before widespread use of highly-active antiretroviral therapy: a collaborative re-analysis. *Lancet* 2000; **355(9210)**:1131-1137.

14. Phillips AN, Pillay D, Miners AH, Bennett DE, Gilks CF, Lundgren JD. Outcomes from monitoring of patients on antiretroviral therapy in resource-limited settings with viral load, CD4 cell count, or clinical observation alone: a computer simulation model. *Lancet* 2008; **371(9622)**:1443-1451.

15. Koot M, Keet IPM, Vos AHV, Degoede REY, Roos MTL, Coutinho RA*, et al.* Prognostic value of HIV-1 syncytium-inducing phenotype for rate of CD4+ cell depletion and progression to AIDS. *Ann Intern Med* 1993; **118(9)**:681-688.

16. BG Gazzard on behalf of the BHIVA Treatment Guidelines Writing Group. British HIV Association guidelines for the treatment of HIV-1-infected adults with antiretroviral therapy 2008. *HIV Med* 2008; **9(8)**:563-608.

17. Clumeck N, Pozniak A, Raffi F, the EACS Executive Committee. European AIDS Clinical Society (EACS) guidelines for the clinical management and treatment of HIV-infected adults. *HIV Med* 2008; **9(2)**:65-71.

18. European AIDS Clinical Society (EACS). EACS Guidelines. October 2013. [www.eacsociety.org](http://www.eacsociety.org). In: 2013.

19. Horne R, Cooper V, Fisher M. Initiation of therapy with a subcutaneously administered antiretroviral in treatment-experienced HIV-infected patients: understanding physician and patient perspectives. *AIDS Care* 2008; **20(9)**:1029-1038.

20. d'Arminio Monforte A, Cozzi-Lepri A, Phillips A, De Luca A, Murri R, Mussini C*, et al.* Interruption of highly active antiretroviral therapy in HIV clinical practice - Results from the Italian cohort of antiretroviral-naive patients. *J Acquir Immune Defic Syndr* 2005; **38(4)**:407-416.

21. Li XH, Margolick JB, Conover CS, Badri S, Riddler SA, Witt MD*, et al.* Interruption and discontinuation of highly active antiretroviral therapy in the multicenter AIDS cohort study. *J Acquir Immune Defic Syndr* 2005; **38(3)**:320-328.

22. Mocroft A, Youle M, Moore A, Sabin CA, Madge S, Lepri AC*, et al.* Reasons for modification and discontinuation of antiretrovirals: results from a single treatment centre. *AIDS* 2001; **15(2)**:185-194.

23. Cambiano V, Lampe FC, Rodger AJ, Smith CJ, Geretti AM, Lodwick RK*, et al.* Long-term trends in adherence to antiretroviral therapy from start of HAART. *AIDS* 2010; **24(8)**:1153-1162.

24. Røge BT, Barfod TS, Kirk O, Katzenstein TL, Obel N, Nielsen H*, et al.* Resistance profiles and adherence at primary virological failure in three different highly active antiretroviral therapy regimens: analysis of failure rates in a randomized study. *HIV Med* 2004; **5(5)**:344-351.

25. Walsh JC, Pozniak AL, Nelson MR, Mandalia S, Gazzard BG. Virologic rebound on HAART in the context of low treatment adherence is associated with a low prevalence of antiretroviral drug resistance. *J Acquir Immune Defic Syndr* 2002; **30(3)**:278-287.

26. Bangsberg DR, Moss AR, Deeks SG. Paradoxes of adherence and drug resistance to HIV antiretroviral therapy. *J Antimicrob Chem* 2004; **53(5)**:696-699.

27. Paterson DL, Swindells S, Mohr J, Brester M, Vergis EN, Squier C*, et al.* Adherence to protease inhibitor therapy and outcomes in patients with HIV infection. *Ann Intern Med* 2000; **133(1)**:21-30.

28. Nieuwkerk P, Gisolf E, Sprangers M, Danner S, Prometheus SG. Adherence over 48 weeks in an antiretroviral clinical trial: variable within patients, affected by toxicities and independently predictive of virological response. *Antivir Ther* 2001; **6(2)**:97-103.

29. Carrieri MP, Raffi F, Lewden C, Sobel A, Michelet C, Cailleton V*, et al.* Impact of early versus late adherence to highly active antiretroviral therapy on immuno-virological response: a 3-year follow-up study. *Antivir Ther* 2003; **8(6)**:585-594.

30. Walsh JC, Mandalia S, Gazzard BG. Responses to a 1 month self-report on adherence to antiretroviral therapy are consistent with electronic data and virological treatment outcome. *AIDS* 2002; **16(2)**:269-277.

31. Bangsberg DR, Porco TC, Kagay C, Charlebois ED, Deeks SG, Guzman D*, et al.* Modeling the HIV protease inhibitor adherence-resistance curve by use of empirically derived estimates. *J Infect Dis* 2004; **190(1)**:162-165.

32. Bannister WP, Kirk O, Gatell JM, Knysz B, Viard JP, Mens H*, et al.* Regional changes over time in initial virologic response rates to combination antiretroviral therapy across Europe. *J Acquir Immune Defic Syndr* 2011; **42(2)**:229-237.

33. Loveday C, Lampe F, Youle M, Tyrer M, Madge S, Sabin CA*, et al.* Potential for transmission of resistant virus: estimation of the proportion of treated people with resistant virus and viral load > 400 cps/mL. In: *10th Conference on Retroviruses and Opportunistic Infections*; 2003.

34. Lampe FC, Gatell JM, Staszewski S, Johnson MA, Pradier C, Gill MJ*, et al.* Changes over time in risk of initial virological failure of combination antiretroviral therapy - A multicohort analysis, 1996 to 2002. *Arch Intern Med* 2006; **166(5)**:521-528.

35. The Collaboration of Observational HIV Epidemiological Research Europe (COHERE) study group. Response to combination antiretroviral therapy: variation by age. *AIDS* 2008; **22(12)**:1463-1473.

36. Staszewski S, Miller V, Sabin C, Schlecht C, Gute P, Stamm S*, et al.* Determinants of sustainable CD4 lymphocyte count increases in response to antiretroviral therapy. *AIDS* 1999; **13(8)**:951-956.

37. Ledergerber B, Egger M, Opravil M, Telenti A, Hirschel B, Battegay M*, et al.* Clinical progression and virological failure on highly active antiretroviral therapy in HIV-1 patients: a prospective cohort study. *Lancet* 1999; **353(9156)**:863-868.

38. Gallant JE, Staszewski S, Pozniak AL, DeJesus E, Suleiman JMAH, Miller MD*, et al.* Efficacy and safety of tenofovir DF vs stavudine in combination therapy in antiretroviral-naive patients - A 3-year randomized trial. *JAMA* 2004; **292(2)**:191-201.

39. The UK Collaborative Group on HIV Drug Resistance and UK CHIC Study Group. Long term probability of detection of HIV-1 drug resistance after starting antiretroviral therapy in routine clinical practice. *AIDS* 2005; **19(5)**:487-494.

40. Harrigan PR, Hogg RS, Dong WWY, Yip B, Wynhoven B, Woodward J*, et al.* Predictors of HIV drug-resistance mutations in a large antiretroviral-naive cohort initiating triple antiretroviral therapy. *J Infect Dis* 2005; **191(3)**:339-347.

41. Phillips AN, Staszewski S, Weber R, Kirk O, Francioli P, Miller V*, et al.* HIV viral load response to antiretroviral therapy according to the baseline CD4 cell count and viral load. *JAMA* 2001; **286(20)**:2560-2567.

42. Staszewski S, Miller V, Sabin C, Carlebach A, Berger AM, Weidmann E*, et al.* Virological response to protease inhibitor therapy in an HIV clinic cohort. *AIDS* 1999; **13(3)**:367-373.

43. van Leth F, Phanuphak P, Ruxrungtham K, Baraldi E, Miller S, Gazzard B*, et al.* Comparison of first-line antiretroviral therapy with regimens including nevirapine, efavirenz, or both drugs, plus stavudine and lamivudine: a randomised open-label trial, the 2NN Study. *Lancet* 2004; **363(9417)**:1253-1263.

44. Bofill M, Janossy G, Lee CA, Macdonaldburns D, Phillips AN, Sabin C*, et al.* Laboratory control values for CD4 and CD8 T lymphocytes. Implications for HIV-1 diagnosis. *Clin Exp Immunol* 1992; **88(2)**:243-252.

45. Maini MK, Gilson RJC, Chavda N, Gill S, Fakoya A, Ross EJ*, et al.* Reference ranges and sources of variability of CD4 counts in HIV-seronegative women and men. *Genitourinary Medicine* 1996; **72(1)**:27-31.

46. Mocroft A, Phillips AN, Gatell J, Ledergerber B, Fisher M, Clumeck N*, et al.* Normalisation of CD4 counts in patients with HIV-1 infection and maximum virological suppression who are taking combination antiretroviral therapy: an observational cohort study. *Lancet* 2007; **370(9585)**:407-413.

47. The PLATO Collaboration. Predictors of trend in CD4-positive T-cell count and mortality among HIV-1-infected individuals with virological failure to all three antiretroviral-drug classes. *Lancet* 2004; **364(9428)**:51-62.

48. Viard JP, Mocroft A, Chiesi A, Kirk O, Roge B, Panos G*, et al.* Influence of age on CD4 cell recovery in human immunodeficiency virus-infected patients receiving highly active antiretroviral therapy: Evidence from the EuroSIDA study. *J Infect Dis* 2001; **183(8)**:1290-1294.

49. Moore RD, Keruly JC. CD4+ cell count 6 years after commencement of highly active antiretroviral therapy in persons with sustained virologic suppression. *Clin Infect Dis* 2007; **44(3)**:441-446.

50. Bennett KK, DeGruttola VG, Marschner IC, Havlir DV, Richman DD. Baseline predictors of CD4 T-lymphocyte recovery with combination antiretroviral therapy. *Jaids-Journal of Acquired Immune Deficiency Syndromes* 2002; **31(1)**:20-26.

51. Thiebaut R, Pellegrin I, Chene G, Viallard JF, Fleury H, Moreau JF*, et al.* Immunological markers after long-term treatment interruption in chronically HIV-1 infected patients with CD4 cell count above 400x10(6) cells/I. *AIDS* 2005; **19(1)**:53-61.

52. Boschi A, Tinelli C, Ortolani P, Moscatelli G, Morigi G, Arlotti M. CD4+cell-count-guided treatment interruptions in chronic HIV-infected patients with good response to highly active antiretroviral therapy. *AIDS* 2004; **18(18)**:2381-2389.

53. Tebas P, Henry K, Mondy K, Deeks S, Valdez H, Cohen C*, et al.* Effect of prolonged discontinuation of successful antiretroviral therapy on CD4+ T cell decline in human immunodeficiency virus-infected patients: Implications for intermittent therapeutic strategies. *J Infect Dis* 2002; **186(6)**:851-854.

54. Youle M, Janossy G, Turnbull W, Tilling R, Loveday C, Mocroft A*, et al.* Changes in CD4 lymphocyte counts after interruption of therapy in patients with viral failure on protease inhibitor-containing regimens. *AIDS* 2000; **14(12)**:1717-1720.

55. Skiest DJ, Morrow P, Allen B, McKinsey J, Crosby C, Foster B*, et al.* It is safe to stop antiretroviral therapy in patients with preantiretroviral CD4 cell counts > 250 cells/μL. *J Acquir Immune Defic Syndr* 2004; **37(3)**:1351-1357.

56. Lawrence J, Mayers DL, Hullsiek KH, Collins G, Abrams DI, Reisler RB*, et al.* Structured treatment interruption in patients with multidrug-resistant human immunodeficiency virus. *N Engl J Med* 2003; **349(9)**:837-846.

57. Fischer M, Hafner R, Schneider C, Trkola A, Joos B, Joller H*, et al.* HIV RNA in plasma rebounds within days during structured treatment interruptions. *AIDS* 2003; **17(2)**:195-199.

58. Achenbach CJ, Till M, Palella FJ, Knoll MD, Terp SM, Kalnins AU*, et al.* Extended antiretroviral treatment interruption in HIV-infected patients with long-term suppression of plasma HIV RNA. *HIV Med* 2005; **6(1)**:7-12.

59. Wit FWNM, Blanckenberg DH, Brinkman K, Prins JM, van der Ende ME, Schneider MME*, et al.* Safety of long-term interruption of successful antiretroviral therapy: the ATHENA cohort study. *AIDS* 2005; **19(3)**:345-348.

60. Johnson VA, Calvez V, Gunthard HF, Paredes R, Pillay D, Shafer RW*, et al.* Update of the drug resistance mutations in HIV-1: March 2013. *Top Antivir Med* 2013; **21(1)**:6-14.

61. Walter H, Low P, Harrer T, Schmitt M, Schwingel E, Tschochner M*, et al.* No evidence for persistence of multidrug-resistant viral strains after a 7-month treatment interruption in an HIV-1-infected individual. *J Acquir Immune Defic Syndr* 2002; **31(2)**:137-146.

62. Devereux HL, Emery VC, Johnson MA, Loveday C. Replicative fitness in vivo of HIV-1 variants with multiple drug resistance-associated mutations. *J Med Virol* 2001; **65(2)**:218-224.

63. Deeks SG, Grant RM, Wrin T, Paxinos EE, Liegler T, Hoh R*, et al.* Persistence of drug-resistant HIV-1 after a structured treatment interruption and its impact on treatment response. *AIDS* 2003; **17(3)**:361-370.

64. Birk M, Svedhem V, Sonnerborg A. Kinetics of HIV-1 RNA and resistance-associated mutations after cessation of antiretroviral combination therapy. *AIDS* 2001; **15(11)**:1359-1368.

65. Hance AJ, Lemiale V, Izopet J, Lecossier D, Joly V, Massip P*, et al.* Changes in human immunodeficiency virus type 1 populations after treatment interruption in patients failing antiretroviral therapy. *J Virol* 2001; **75(14)**:6410-6417.

66. Tarwater PM, Parish M, Gallant JE. Prolonged treatment interruption after immunologic response to highly active antiretroviral therapy. *Clin Infect Dis* 2003; **37(11)**:1541-1548.

67. CASCADE Collaboration. Short-term risk of AIDS according to current CD4 cell count and viral load in antiretroviral drug-naive individuals and those treated in the monotherapy era. *AIDS* 2004; **18(1)**:51-58.

68. Phillips AN, Lee CA, Elford J, Webster A, Janossy G, Timms A*, et al.* More rapid progression to AIDS in older HIV-infected people - the role of CD4+ T-cell counts. *J Acquir Immune Defic Syndr* 1991; **4(10)**:970-975.

69. Touloumi G, Hatzakis A, Rosenberg PS, O'Brien TR, Goedert JJ. Effects of age at seroconversion and baseline HIV RNA level on the loss of CD4+ cells among persons with hemophilia. *AIDS* 1998; **12(13)**:1691-1697.

70. Mallolas J, Zamora L, Gatell JM, Miro JM, Vernet E, Valls ME*, et al.* Primary Prophylaxis for Pneumocystis-Carinii Pneumonia - A Randomized Trial Comparing Cotrimoxazole, Aerosolized Pentamidine and Dapsone Plus Pyrimethamine. *AIDS* 1993; **7(1)**:59-64.

71. Phillips AN, Elford J, Sabin C, Bofill M, Janossy G, Lee CA. Immunodeficiency and the risk of death in HIV infection. *JAMA* 1992; **268(19)**:2662-2666.

72. Touloumi G, Karafoulidou A, Gialeraki A, Katsarou O, Milona I, Kapsimali V*, et al.* Determinants of progression of HIV infection in a Greek hemophilia cohort followed for up to 16 years after seroconversion. *J Acquir Immune Defic Syndr* 1998; **19(1)**:89-97.

73. Lundgren JD, Pedersen C, Clumeck N, Gatell JM, Johnson AM, Ledergerber B*, et al.* Survival differences in European patients with AIDS, 1979−89. *BMJ* 1994; **308(6936)**:1068-1073.

74. Antiretroviral Therapy Cohort Collaboration (ART-CC). Variable impact on mortality of AIDS-defining events diagnosed during combination antiretroviral therapy: not all AIDS-defining conditions are created equal. *Clin Infect Dis* 2009; **48(8)**:1138-1151.

75. Deeks SG, Phillips AN. HIV infection, antiretroviral treatment, ageing, and non-AIDS related morbidity. *BMJ* 2009; **338**:a3172.

76. Phillips AN, Neaton J, Lundgren JD. The role of HIV in serious diseases other than AIDS. *AIDS* 2008; **22(18)**:2409-2418.

77. Frisch M, Biggar RJ, Engels EA, Goedert JJ, for the AIDS-Cancer Match Registry Study Group. Association of cancer with AIDS-related immunosuppression in adults. *JAMA* 2001; **285(13)**:1736-1745.

78. Herida M, Mary-Krause M, Kaphan R+, Cadranel J, Poizot-Martin I, Rabaud C*, et al.* Incidence of non-AIDS-defining cancers before and during the highly active antiretroviral therapy era in a cohort of human immunodeficiency virus-infected patients. *J Clin Oncol* 2003; **21(18)**:3447-3453.

79. Maggi P, Quirino T, Ricci E, De Socio GVL, Gadaleta A, Ingrassia F*, et al.* Cardiovascular Risk Assessment in Antiretroviral-Naive HIV Patients. *AIDS Patient Care STDS* 2009; **23(10)**:809-813.

80. Francisci D, Giannini S, Baldelli F, Leone M, Belfiori B, Guglielmini G*, et al.* HIV type 1 infection, and not short-term HAART, induces endothelial dysfunction. *AIDS* 2009; **23(5)**:589-596.

81. Lewden C, Chene G, Morlat P, Raffi F, Dupon M, Dellamonica P*, et al.* HIV-infected adults with a CD4 cell count greater than 500 cells/mm^3^ on long-term combination antiretroviral therapy reach same mortality rates as the general population. *J Acquir Immune Defic Syndr* 2007; **46(1)**:72-77.

82. Study Group on Death Rates at High CD4 Count in Antiretroviral Naive Patients. Death rates in HIV-positive antiretroviral-naive patients with CD4 count greater than 350 cells per mu L in Europe and North America: a pooled cohort observational study. *Lancet* 2010; **376(9738)**:340-345.

83. Kuller LH, Ockene JK, Meilahn E, Wentworth DN, Svendsen KH, Neaton JD. Cigarette-Smoking and Mortality. *Preventive Medicine* 1991; **20(5)**:638-654.

84. Lodi S, Phillips AN, Touloumi G, Geskus RB, Meyer L, Thiebaut R*, et al.* Time from Human Immunodeficiency Virus seroconversion to reaching CD4+ cell count thresholds of <200, <350 and <500 cells/mm^3^: Assessment of need following changes in treatment guidelines. *Clin Infect Dis* 2011; **53(8)**:817-825.

85. Dorrucci M, Rezza G, Porter K, Phillips A. Temporal trends in postseroconversion CD4 cell count and HIV load: The Concerted Action on Seroconversion to AIDS and Death in Europe Collaboration, 1985-2002. *J Infect Dis* 2007; **195(4)**:525-534.

86. Egger M, May M, Chene G, Phillips AN, Ledergerber B, Dabis F*, et al.* Prognosis of HIV-1-infected patients starting highly active antiretroviral therapy: a collaborative analysis of prospective studies. *Lancet* 2002; **360(9327)**:119-129.

87. The UK Collaborative Group on HIV Drug Resistance and UK CHIC Study Group. Long term probability of detecting drug-resistant HIV in treatment-naive patients initiating combination antiretroviral therapy. *Clin Infect Dis* 2010; **50(9)**:1275-1285.

88. Sterne JAC, Hernan MA, Ledergerber B, Tilling K, Weber R, Sendi P*, et al.* Long-term effectiveness of potent antiretroviral therapy in preventing AIDS and death: a prospective cohort study. *Lancet* 2005; **366(9483)**:378-384.

89. Smith CJ, Phillips AN, Hill T, Fisher M, Gazzard B, Porter K*, et al.* The rate of viral rebound after attainment of an HIV load < 50 copies/mL according to specific antiretroviral drugs in use: Results from a multicenter cohort study. *J Infect Dis* 2005; **192(8)**:1387-1397.

90. Mocroft A, Phillips AN, Soriano V, Rockstroh J, Blaxhult A, Katlama C*, et al.* Reasons for stopping antiretrovirals used in an initial highly active antiretroviral regimen: Increased incidence of stopping due to toxicity or patient/physician choice in patients with hepatitis C coinfection. *AIDS Res Hum Retroviruses* 2005; **21(9)**:743-752.

91. Mocroft A, Ledergerber B, Viard JP, Staszewski S, Murphy M, Chiesi A*, et al.* Time to virological failure of 3 classes of antiretrovirals after initiation of highly active antiretroviral therapy: Results from the EuroSIDA Study Group. *J Infect Dis* 2004; **190(11)**:1947-1956.

92. The Pursuing Later Treatment Options II (PLATO II) Project Team for the Collaboration of Observational HIV Epidemiological Research Europe (COHERE). Triple-Class Virologic Failure in HIV-Infected Patients Undergoing Antiretroviral Therapy for Up to 10 Years. *Arch Intern Med* 2010; **170(5)**:410-419.

93. Grover D, Copas A, Green H, Edwards SG, Dunn DT, Sabin C*, et al.* What is the risk of mortality following diagnosis of multidrug-resistant HIV-1? *J Antimicrob Chem* 2008; **61(3)**:705-713.
